# Nitric oxide and peroxynitrite trigger and enhance release of neutrophil extracellular traps

**DOI:** 10.1007/s00018-019-03331-x

**Published:** 2019-10-24

**Authors:** Aneta Manda-Handzlik, Weronika Bystrzycka, Adrianna Cieloch, Eliza Glodkowska-Mrowka, Ewa Jankowska-Steifer, Edyta Heropolitanska-Pliszka, Agnieszka Skrobot, Angelika Muchowicz, Olga Ciepiela, Malgorzata Wachowska, Urszula Demkow

**Affiliations:** 1 Department of Laboratory Medicine and Clinical Immunology of Developmental Age, Medical University of Warsaw, Zwirki i Wigury 63a Street, 02-091 Warsaw, Poland; 2 Postgraduate School of Molecular Medicine, Medical University of Warsaw, Zwirki i Wigury 61 Street, 02-091 Warsaw, Poland; 3 The Finsen Laboratory, Faculty of Health Sciences, Rigshospitalet, University of Copenhagen, Ole Maaloesvej 5, 2200 Copenhagen, Denmark; 4 Biotech Research and Innovation Centre (BRIC), University of Copenhagen, Ole Maaloesvej 5, 2200 Copenhagen, Denmark; 5 Faculty of Health Sciences, Danish Stem Cell Centre (DanStem), University of Copenhagen, Ole Maaloesvej 5, 2200 Copenhagen, Denmark; 6 Department of Biology, The Bioinformatics Centre, University of Copenhagen, Ole Maaloesvej 5, 2200 Copenhagen, Denmark; 7 Department of Histology and Embryology, Medical University of Warsaw, Chalubinskiego 5 Street, 02-004 Warsaw, Poland; 8 Department of Immunology, The Children’s Memorial Health Institute, Aleja Dzieci Polskich 20, 04-730 Warsaw, Poland; 9 Department of Immunology, Medical University of Warsaw, Jana Nielubowicza 5 Street, 02-097 Warsaw, Poland; 10 Department of Laboratory Diagnostics, Medical University of Warsaw, Banacha 1a Street, 02-097 Warsaw, Poland

**Keywords:** Autophagy, Neutrophil extracellular traps, Nitric oxide, Peroxynitrite, Phosphoinositide 3-kinases, Reactive nitrogen species

## Abstract

Despite great interest, the mechanism of neutrophil extracellular traps (NETs) release is not fully understood and some aspects of this process, e.g. the role of reactive nitrogen species (RNS), still remain unclear. Therefore, our aim was to investigate the mechanisms underlying RNS-induced formation of NETs and contribution of RNS to NETs release triggered by various physiological and synthetic stimuli. The involvement of RNS in NETs formation was studied in primary human neutrophils and differentiated human promyelocytic leukemia cells (HL-60 cells). RNS (peroxynitrite and nitric oxide) efficiently induced NETs release and potentiated NETs-inducing properties of platelet activating factor and lipopolysaccharide. RNS-induced NETs formation was independent of autophagy and histone citrullination, but dependent on the activity of phosphoinositide 3-kinases (PI3K) and myeloperoxidase, as well as selective degradation of histones H2A and H2B by neutrophil elastase. Additionally, NADPH oxidase activity was required to release NETs upon stimulation with NO, as shown in NADPH-deficient neutrophils isolated from patients with chronic granulomatous disease. The role of RNS was further supported by increased RNS synthesis upon stimulation of NETs release with phorbol 12-myristate 13-acetate and calcium ionophore A23187. Scavenging or inhibition of RNS formation diminished NETs release triggered by these stimuli while scavenging of peroxynitrite inhibited NO-induced NETs formation. Our data suggest that RNS may act as mediators and inducers of NETs release. These processes are PI3K-dependent and ROS-dependent. Since inflammatory reactions are often accompanied by nitrosative stress and NETs formation, our studies shed a new light on possible mechanisms engaged in various immune-mediated conditions.

**Electronic supplementary material:**

The online version of this article (10.1007/s00018-019-03331-x) contains supplementary material, which is available to authorized users.

## Introduction

Neutrophils are effector cells of the innate immune system responsible for the defense against invading pathogens upon recruitment to the site of infection, injury or inflammation. After migration to the tissue, neutrophils are able to destroy pathogens through several mechanisms, including the release of neutrophil extracellular traps (NETs) [1]. These antimicrobial structures are composed of DNA complexed with histones as well as granular and cytoplasmic proteins, such as neutrophil elastase (NE), myeloperoxidase (MPO), and cathepsin G. Most studies point to nucleus as the source of NETs-bound DNA, however, the release of mitochondrial DNA has also been described [2]. In physiological conditions, these DNA-containing traps immobilize and kill microorganisms, serving as an important mechanism to control bacterial, fungal, parasitic, and viral infections. Since NETs contain a magnitude of proteolytically active proteins, an uncontrolled NETs formation process may promote tissue damage. Indeed, imbalance between NETs formation and clearance can contribute to multiple pathological conditions such as systemic lupus erythematosus, thrombosis, cystic fibrosis or even formation of cancer metastases, being “a double-edged sword” [2, 3].

Various processes have been associated with NETs release, including citrullination of histones, autophagy, the formation of reactive oxygen species (ROS), and activation of protein kinase B (AKT) and mitogen-activated protein kinases [MAPK, namely: p-38 and extracellular signal–regulated kinase (ERK)] [2, 4]. Current consensus implies that depending on a stimulus, different molecular events may be involved in this process [5]. For example, histone citrullination is induced only by some classical inducers of NETs formation, such as calcium ionophores, and remains controversial for others, including phorbol 12-myristate 13-acetate (PMA) [6]. Furthermore, some stimuli, including PMA, granulocyte/macrophage colony-stimulating factor and complement factor 5a, and *E. coli* or *P. aeruginosa* bacteria, require synthesis of ROS by NADPH oxidase to induce NETs release [7–9]. Others, such as calcium ionophore A23187 (CI) or antigen–antibody complexes, require mitochondrial ROS formation [2, 4]. First evidence on the indispensability of ROS came from the studies on neutrophils isolated from patients suffering from chronic granulomatous disease (CGD), who are unable to produce superoxide due to inherited deficiency of NADPH oxidase [7]. It was shown that ROS are necessary to induce translocation of NE from azurophilic granules to the nucleus, where it degrades histones and promotes chromatin decondensation [10].

Under inflammatory conditions, production of ROS is tightly correlated to the generation of another group of redox signaling molecules—reactive nitrogen species (RNS) [11]. RNS are derived from nitric oxide (NO), a product of nitric oxide synthase activity. The fate of NO in biological systems is controlled by three main processes—NO diffusion and intracellular consumption, autooxidation to nitrogen trioxide (N_2_O_3_), and highly efficient reaction with superoxide (O_2_^•−^, with several enzymatic sources, including NADPH oxidase), which yields peroxynitrite (ONOO^−^) [12, 13]. Peroxynitrite, in equilibrium with peroxynitrous acid, subsequently may react with carbon dioxide and give rise to various ROS and RNS: nitrogen dioxide (NO_2_), carbonate radical (CO_3_^•–^), and hydroxyl radical (•OH) [14].

Both ROS and RNS are crucial for normal function of the immune system, since they are engaged in the killing of invading pathogens and in the regulation of immune response [15]. So far, the studies deciphering mechanisms of NETs formation focused mostly on the role of ROS and our understanding of RNS contribution to NETs formation is largely limited [16, 17]. Early studies by Patel et al. suggested the potential role of NO as NETs inducer via its modulation of ROS production [16]. Yet, the influence of RNS on other pathways and key molecules involved in the release of NETs, the ability of NO metabolites to induce NETs, as well as the contribution of RNS to NETs formation triggered by other stimuli, remain unclear.

As the relationship between RNS and NETs awaits to be elucidated, the aim of our study was to shed a light on the mechanisms underlying RNS-induced formation of NETs and to investigate whether RNS contribute to NETs release triggered by various physiological and synthetic stimuli. In this study, we specifically focused on the role of the following RNS: NO and ONOO^−^.

## Materials and methods

### Sources of granulocytes and granulocyte-like cells

For most experiments, neutrophils were isolated from peripheral blood samples or buffy coats purchased from a Regional Blood Donation Center. In addition to the blood sampled from healthy adult blood donors, peripheral blood was collected from nine CGD patients (including five children) and from six healthy children which served as controls (Supplementary Tables 1 and 2). Diagnosis of CGD was made based on clinical history and impaired oxidative burst assessed by flow cytometry dihydrorhodamine (DHR) 123 oxidation assay and/or nitroblue tetrazolium (NBT) assay. At the time of the sampling, CGD patients were free of acute infections. In accordance with local law, each adult blood donor gave the blood donation center a written permission to sell their blood samples/constituents for scientific purposes. In other cases, an informed, written consent was signed by each individual or their caretakers.

HL-60 cell culture propagation and differentiation with dimethylformamide (DMF) into granulocyte-like cells were performed exactly as described previously [18].

The study design was approved by local Ethics Committee (reference numbers: KB/225/2014 and KB/55/A/2017).

### Neutrophil isolation and stimulation

Neutrophils were isolated by gradient density centrifugation and sedimentation in 1% polyvinyl alcohol, as previously described [19]. To stimulate NETs release, the cells were suspended in RPMI-1640 without phenol red with 10 mM HEPES and seeded at 2.5 × 10^4^ cells per well into 8-well Lab-Tek chambers for NETs immunolabeling; 0.5–1 × 10^5^ cells per well were seeded into 24- or 96-well plates for extracellular DNA quantification; 2–5 × 10^4^ cells per well were seeded into 24- or 48- well plates for live NETs imaging. The cells were allowed to settle for 30 min at 37 °C, 5% CO_2_ in the presence or absence of various inhibitors (Supplementary Fig. 1), as indicated in Figure legends. Next, the cells were stimulated with: 100–500 µM S-nitroso-N-acetyl-D,L-penicillamine (SNAP, NO donor), 50–200 µM sodium peroxynitrite, 2.5 µM platelet activating factor C-16 (PAF, Ref. no: 60900, Cayman chemicals), 2 µg/ml lipopolysaccharide (LPS) isolated from *E.coli* (Ref. no: L2755, Sigma-Aldrich), 10 µg/ml LPS isolated from *P. aeruginosa* (Ref. no: L9143, Sigma-Aldrich), 100 nM PMA, 4 µM CI, 100 ng/ml tumor necrosis factor α (TNF-α), or 100 ng/ml interleukin 8 (IL-8) for an indicated time. Unstimulated cells served as control.

### NETs immunostaining

At indicated time points, the cells were fixed with 4% paraformaldehyde for 20 min, permeabilized with 0.1% of Triton X-100, and blocked with 10% goat serum with 5% bovine serum albumin (BSA). The samples were stained with anti-NE antibodies (ab21595, 1:100, overnight, 4 °C) followed by secondary antibodies conjugated with FITC (ab6717, 1:2000, 1 h, room temperature, RT), and DNA was counterstained with Hoechst 33342. Alternatively, the samples were blocked with 1% BSA, stained with antibodies directed against MPO (ab11729, 1:500, overnight, 4 °C), and DNA was counterstained with SYTOX Orange. All antibodies for immunostaining were purchased from Abcam (Cambridge, UK). Slides were assessed with Leica DMi8 fluorescent microscope equipped with a 40× and a 10× magnification objectives (Leica, Wetzlar, Germany) or with Zeiss Axio Observer Z1 confocal microscope equipped with EC Plan-Neofluar 40× oil objective (Carl Zeiss AG, Oberkochen, Germany).

### Live cells imaging

NETs formation was assessed in unfixed samples by staining with DNA-binding dyes. SYTOX Green was used to visualize cells with compromised cell membranes, releasing NETs and Hoechst 33342 was used to visualize and quantitate all cells. In samples where Hoechst 33342 was not used, total number of cells was assessed based on transient-light images. At least ten images were taken at 40× magnification with Leica DMi8 microscope; in samples stained solely with SYTOX Green, routinely 250–500 cells were assessed per condition, whereas in samples stained with SYTOX Green and Hoechst 33342, at least 100 cells were assessed per condition.

To quantitate nuclear decondensation, nuclei areas were measured using ImageJ software v. 1.50i (National Institute of Health, Bethesda, MD, USA) [20]. Analysis of particles was performed after manually adjusting the color threshold, so that the whole area stained with DNA-binding dye was measured. Alternatively, e.g. when multiple objects overlapped, nuclei areas were measured after drawing regions of interest around objects with one of the drawing tools. This analysis was performed by a single, experienced, unblinded researcher. Subsequently, frequency function was used to obtain a distribution of the number of stained objects vs. the range of nuclear area (Excel, Microsoft, Redmond, WA, USA). The number of stained objects within each nuclear area range was divided by the total number of cells and plotted as the percentage.

**Fig. 1:**
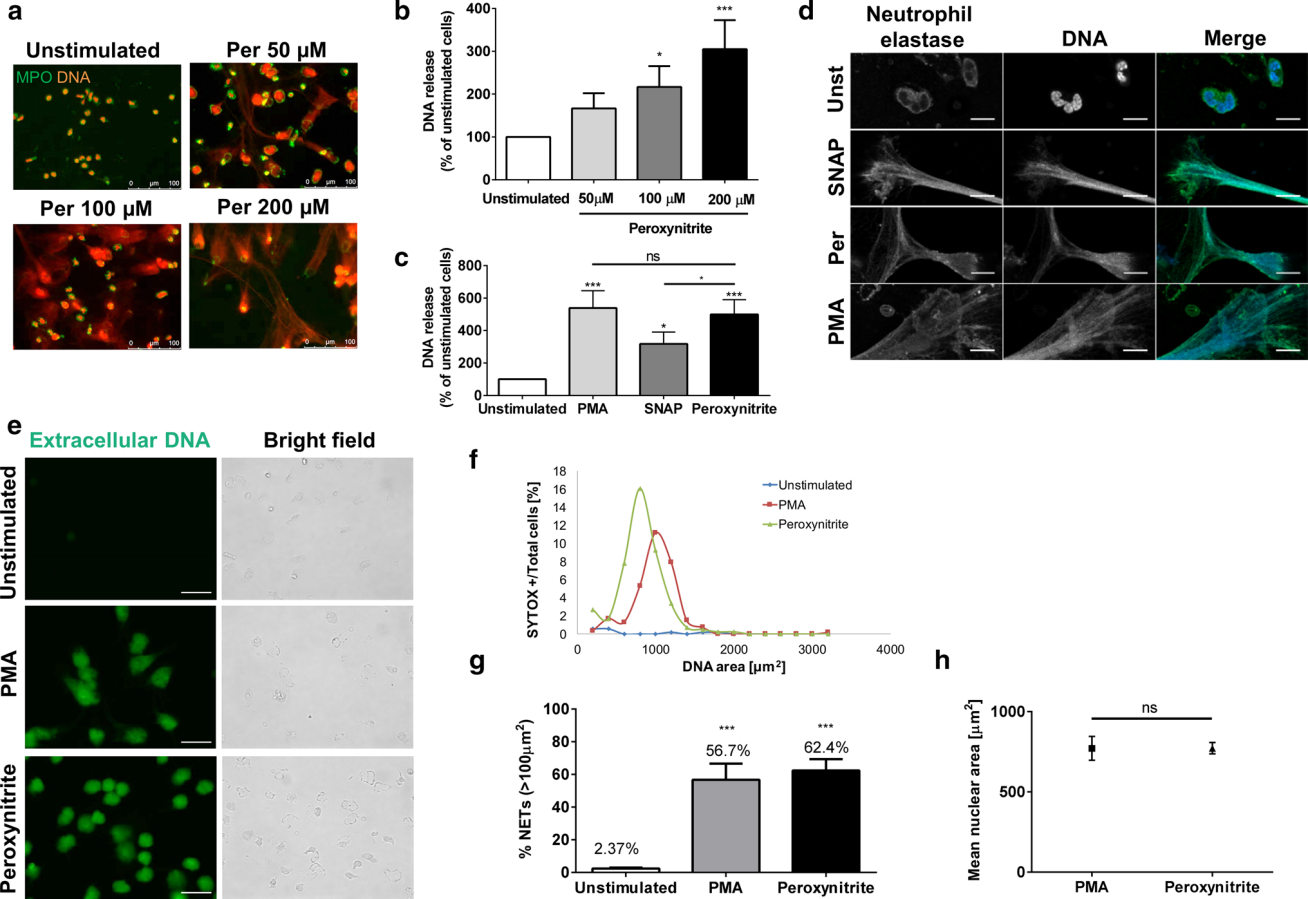
Reactive nitrogen species efficiently stimulate release of neutrophil extracellular traps (NETs). **a**–**h** Neutrophils were stimulated with increasing concentrations of peroxynitrite (Per, **a**, **b**), 100 µM peroxynitrite (**c**–**h**), 500 µM S-nitroso-N-acetyl-D,L-penicillamine (SNAP, **c**, **d**) or 100 nM phorbol 12-myristate 13-acetate (PMA, **c**–**h**) for 3 h. Unstimulated cells served as a control. **a**–**d** NETs formation was assessed after immunolabeling by conventional (**a**) or confocal (**d**) fluorescent microscopy and fluorometric measurement of DNA release (**b**, **c**). **e**–**h** After 3-h stimulation, samples were stained with SYTOX Green, which is impermeable to live cells, and at least ten images were taken at 40× magnification using fluorescent and transient lights. Areas of SYTOX-positive (SYTOX+) objects were measured using ImageJ software. **a**, **d**, **e** Representative images of one out of three (**a**, **d**) or five (**e**) experiments using different blood donors. **f** Distribution of SYTOX+ cells percentage over corresponding DNA area, shown results are representative for one out of five experiments performed using different donors. **g** Comparison of percentage of NET-releasing cells between samples, where NETs were defined as SYTOX+ objects of area larger than 100 µm^2^. **h** Comparison of the degree of nuclear decondensation (area of SYTOX+ objects) between peroxynitrite-stimulated and PMA-stimulated samples. **b**, **c**, **g** Results are shown as means + SEM and were analyzed by one-way ANOVA with post hoc Dunn’s (**b**, **c**) or Dunnett’s (**g**) test vs. unstimulated cells unless marked otherwise; *n* = 10 (**b**), *n* = 8 (**c**), *n* = 5 (**g**), where *n* is the number of experiments performed using different blood donors. **h** Results are shown as means with SEM out of five experiments using different donors; *t* test. *(*p* ≤ 0.05), ***(*p* ≤ 0.001). Scale bars represent 10 µm (**d**) and 50 µm (**e**)

In some experiments (i.e. Fig. 1g and Supplementary Fig. 2c), measurements of nuclear area of SYTOX-positive objects performed with ImageJ were used to quantify NETs release. NETs were defined as SYTOX-positive events of area larger than 100 μm^2^, assuming that nuclear area of unstimulated granulocyte is ~ 80 μm^2^ [21, 22]. Subsequently, the number of SYTOX-positive events of area > 100 μm^2^ was divided by the total number of cells, as manually counted in bright field images. The results were presented as the percentage of cells releasing NETs.

### Measurement of extracellular DNA release

To quantify NETs release after 3-h stimulation, extracellular DNA was detached with 500 mIU/ml MNase for 20 min at 37 °C. MNase activity was then stopped with 5 mM EDTA. Plates were centrifuged (10 min, 800 g) and the supernatant was transferred in triplicates to black 96-well plates and DNA release was measured fluorometrically after the addition of 200 nM SYTOX Green in a FLUOstar OMEGA plate reader (BMG Labtech, Ortenberg, Germany).

If not stated otherwise, the data were normalized to DNA release in an unstimulated sample. To that end, fluorescence readouts in all conditions were divided by readout from an unstimulated sample and the results were shown in percentage.

### Assessment of reactive oxygen/nitrogen species release

To fluorometrically monitor reactive oxygen species/reactive nitrogen species (ROS/RNS) release, 1 × 10^5^ cells per well were seeded into black 96-well plates. Cells were loaded with 10 µM DAF-FM DA (NO probe) for 1 h at 37 °C, 5% CO_2_, the excess probe was washed away and cells were allowed to complete de-esterification of the probe for following 30 min, in the presence of various inhibitors when necessary. Alternatively, cells were incubated for 30 min with DHR123 in the presence of inhibitors when necessary; excess probe was washed away and the inhibitors were added again to the cells to the final concentration. Then, the cells were stimulated as described in Figure legends and fluorescence was monitored every 15 min for 4 h in the FLUOstar OMEGA plate reader.

To assess ROS production by NBT (nitroblue tetrazolium) assay, 2–2.5 × 10^4^ neutrophils per well were seeded in 48-well plates or Lab-Tek chamber cover slides with 1 mg/ml NBT with or without appropriate inhibitors and incubated for 30 min at 37 °C, 5% CO_2_. Cells were then stimulated for 2 h, fixed with 4% paraformaldehyde, and the percentage of cells with blue formazan deposits was counted by light microscopy using Leica DMi8 microscope.

### Western blot

Neutrophils were treated as described above. In most experiments, 2.5–5 × 10^6^ neutrophils were centrifuged at an indicated time point and lysed in RIPA buffer supplemented with a protease inhibitor cocktail. Lysates were sonicated, boiled with 5 × Laemmli buffer (5 min, 95 °C) and equal amounts of protein were separated by SDS-PAGE. For histone degradation experiments, 1.5 × 10^5^ neutrophils were incubated in 96-well plates and at an indicated time point, cells were lysed in 100 µl of 1 × SDS loading buffer and equal volumes of the lysate were loaded on gel and separated by SDS-PAGE. Proteins were transferred to nitrocellulose or PVDF membranes, blocked with 5% BSA or 5% milk, and incubated overnight at 4 °C with primary antibodies: anti-citH3 (ab5103, 1:1000 in 5% milk) purchased from Abcam (Cambridge, UK); anti-H2A (#2578, 1:1000 in 5% BSA), anti-H2B (#2934, 1:1000 in 5% milk), anti-p-Akt (#4058, 1:1000 in 5% BSA), anti-ERK 1/2 (#4696, 1:2000 in 5% milk), anti-p-p38 (#9216, 1:2000 in 5% milk), anti-p38 (#8690, 1:1000 in 5% BSA), or anti-LC3A/B (#4108, 1:1000 in 5% BSA) purchased from Cell Signaling (Beverly, MA, USA); anti-H3 (#07-690, 1:10,000 in 5% milk), anti-H4 (#04-8585, 1:2000–1:10,000), or anti-p-ERK 1/2 (#05-797, 1:1000 in 5% milk) purchased from Merck (Merck, Darmstadt, Germany). This was followed by incubation with secondary anti-rabbit or anti-mouse antibodies conjugated with HRP (#7074 or #7076, 1:2000 in 5% milk, 1 h, RT). Anti-GAPDH and anti-ACTB antibodies conjugated with HRP (G9295, 1:25 000–1:50 000; A3854, 1:50 000–1:100 0000, respectively, 0.5–1 h incubation at RT) purchased from Sigma were used as loading controls. Homemade or commercial enhanced chemiluminescence detection kits (WESTAR ETA C ULTRA 2.0 or WESTAR SUPERNOVA, Cyanagen Srl, Bologna, Italy) were used to detect the protein presence.

### Statistical analysis

Data were analyzed using GraphPad Prism Software v. 6 (GraphPad Software, La Jolla, CA, USA). Due to the size of the tested populations, KS normality test was routinely used. Multiple groups were compared with one-way ANOVA with appropriate post hoc tests and two groups were compared with *t* test, unless otherwise specified. *p* ≤ 0.05 was considered statistically significant. For all experiments in which human neutrophils were used, the number of individual experiments specified in Figure legends refers to biological replicates.

The supplementary materials include additional information on materials and methods.

## Results

### NO and peroxynitrite induce release of NETs

To determine whether peroxynitrite (NO metabolite) can stimulate granulocytes to release NETs, we incubated human neutrophils with its increasing, in vivo achievable, concentrations [23]. We observed that exogenously added peroxynitrite induced NETs formation in a concentration-dependent manner (Fig. 1a, b) with statistical significance for concentrations, 100 and 200 μM. For subsequent experiments, we used 100 µM peroxynitrite to induce NETs. Similarly SNAP, a NO donor, stimulated neutrophils to release NETs (Fig. 1c, d).

In subsequent experiments we investigated NETs formation by confocal fluorescent microscopy and fluorometric measurement of DNA release. In our hands, SNAP turned out to be a less potent NETs inducer than peroxynitrite, whereas NETs-inducing potency of peroxynitrite was similar to this of PMA—a positive control in most of our experiments (Fig. 1c). After a 3-h stimulation, all inducers triggered a formation of elongated, web-like structures, co-localizing NE with DNA (Fig. 1d).

To further investigate NETs-inducing properties of peroxynitrite, we visualized unfixed, stimulated cells stained with SYTOX Green. Live imaging revealed the presence of large numbers of SYTOX-positive objects in peroxynitrite-stimulated and PMA-stimulated samples; distributions of SYTOX-positive objects over their corresponding DNA areas were similar for both stimuli (Fig. 1e, f). By defining NETs as SYTOX-positive objects of area larger than 100 µm^2^, we were able to corroborate that comparable fraction of neutrophils responds to stimulation either with peroxynitrite or with PMA (Fig. 1g). These findings were in line with the results of fluorometric NETs formation assay (Fig. 1c). Furthermore, we did not observe any differences in the mean nuclear area between these groups, which confirms that peroxynitrite is just as effective as PMA in the induction of nuclear decondensation (Fig. 1h). In vivo imaging experiments were also performed for SNAP-stimulated neutrophils (Supplementary Fig. 2a–d), confirming that SNAP effectively induces nuclear decondensation and NETs formation, but is not as efficient in NETs induction as PMA.

### RNS induce NETs in phosphoinositide 3-kinases- and ROS-dependent manner

Next, we investigated the mechanisms involved in NETs formation upon stimulation with NO and peroxynitrite, further referred to as RNS for conciseness. We aimed to perform mechanistic studies in two experimental settings—with the use of HL-60 cell line-derived granulocyte-like cells and primary neutrophils isolated from healthy blood donors. In contrast to human neutrophils, HL-60 cells differentiated with dimethylformamide failed to release NETs upon stimulation with RNS, although they responded vigorously to PMA or CI (Supplementary Fig. 3a, b). Furthermore, we did not observe increased production of NO by differentiated HL-60 cells stimulated to release NETs with PMA or CI (Supplementary Fig. 3c). We also failed to generate genetically modified HL-60 cells overexpressing inducible NOS in a constitutive or tetracycline-inducible manner, even though we were able to overexpress this protein in HEK-293T cells with the same vector system and transduce HL-60 cells with luciferase construct (data not shown). Based on these observations, we concluded that granulocyte-like cells derived from HL-60 cells cannot serve as a model for RNS-mediated NETs formation and further mechanistic experiments were performed solely using primary human granulocytes.

**Fig. 2:**
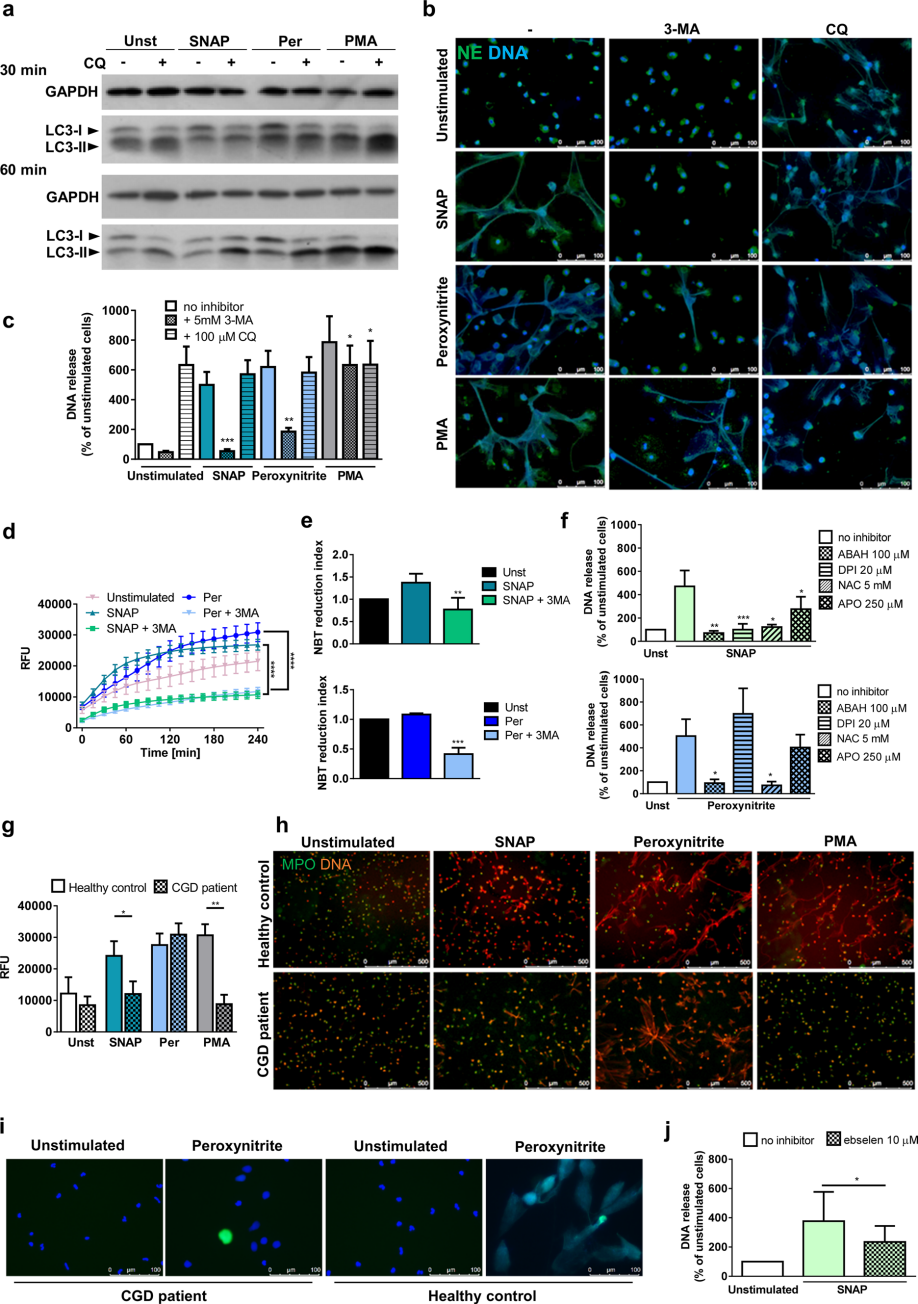
Reactive nitrogen species stimulate NETs in phosphoinositide 3-kinase (PI3K)-dependent and reactive oxygen species (ROS)-dependent manner. **a**–**f**, **j** Neutrophils were preincubated with inhibitors: 100 µM chloroquine (CQ, **a**–**c**), 5 mM 3-methyladenine (3-MA, **b**–**e**), 100 µM 4-aminobenzoic acid hydrazide (ABAH), 20 µM diphenyleneiodonium (DPI), 5 mM N-acetylcysteine (NAC), 250 µM apocynin (APO) (**f**) or 10 µM ebselen (**j**) for 30 min and then stimulated for indicated time with 500 µM SNAP, 100 µM peroxynitrite or 100 nM PMA. **a** After 30- or 60-min stimulation, the cells were lysed and conversion of LC3-I into LC3-II was analyzed in the lysates by western blotting. One representative out of two experiments is shown. **b**, **c**, **f**, **j** NETs release was analyzed after 3-h stimulation microscopically (**b**) and fluorometrically (**c**, **f**, **j**). **d**, **e** To assess production of ROS, prior to stimulation, neutrophils were loaded with dihydrorhodamine 123 and then monitored fluorometrically every 15 min for 4 h post-stimulation (**d**) or with nitroblue tetrazolium (NBT) and blue cells containing formazan deposits were counted under the light microscope after 2-h stimulation; NBT reduction index was calculated by dividing the percentage of blue cells in each condition by the percentage of blue cells in unstimulated samples (**e**). **g**–**i** Neutrophils were isolated from peripheral blood of patients suffering from chronic granulomatous disease (CGD) or healthy controls, stimulated for 3 h and NETs formation was assessed fluorometrically (**g**) and microscopically after immunolabeling (**h**). Alternatively, NETs were visualized after 2-h stimulation by staining with Hoechst 33342 (blue) and SYTOX Green (green) by live imaging; one representative out of two experiments is shown (**i**). **c**, **e**, **f** Results are shown as means + SEM and were analyzed by one-way ANOVA with post hoc Dunnett’s (**c**, **e**, **f—**peroxynitrite) or Dunn’s (**f—**SNAP) test vs. stimulated cells without inhibitor; *n* = 6. **g**, **j** Means + SEM are shown, data were analyzed by *t* tests, *n* = 8 (CGD), *n* = 9 (control) (**g**), *n* = 6 (**j**). **d** Results are shown as means + SEM out of six experiments with different donors and were analyzed by two-way ANOVA with post hoc Bonferroni’s multiple comparisons test. *(*p* ≤ 0.05), **(*p* ≤ 0.01), ***(*p* ≤ 0.001), ****(*p* ≤ 0.0001). *NE* neutrophil elastase, *MPO* myeloperoxidase, *RFU* relative fluorescence units

To uncover molecular mechanisms underlying RNS-induced NETs release, we first examined the role of autophagy (one of the most controversial contributors to NETs formation [5]). Autophagy is a dynamic process, involving sequestration of specific cellular cargo within vesicles called autophagosomes. This is followed by a fusion of autophagosomes with lysosomes, to allow degradation of vesicular content [24]. To analyze, whether peroxynitrite and NO induce autophagy, we assessed autophagosome formation by western blot analysis of the conversion of LC3-I into LC3-II protein and we examined morphological changes characteristic for autophagy by transmission electron microscopy (TEM). In these experiments, samples were incubated in the presence and absence of chloroquine (CQ), as required to determine autophagic flux [24, 25]. Contrary to PMA, neither SNAP nor peroxynitrite induced autophagy in stimulated cells (Fig. 2a, Supplementary Fig. 4).

Interestingly, inhibitors interfering with autophagic sequestration that were also phosphoinositide 3-kinases (PI3K) inhibitors (3-methyladenine (3-MA) and wortmannin), but not specific inhibitors of autophagosome–lysosome fusion (CQ and bafilomycin A1), reduced NETs formation upon RNS stimulation (Fig. 2b, c, Supplementary Fig. 5a, b). PI3K inhibitors completely prevented NETs formation upon SNAP stimulation and were less efficient in the presence of peroxynitrite. Furthermore, 3-MA exerted stronger inhibitory effect than wortmannin in peroxynitrite-stimulated samples (Fig. 2c, Supplementary Fig. 5a). Disruptive effect of wortmannin on peroxynitrite-induced NETs release was confirmed by microscopical analyses. In the presence of wortmannin, a significant proportion of cells retained a decondensed shape instead of forming web-like NETs structures visible in peroxynitrite-stimulated controls (Supplementary Figure 5b). Fluorometric measurements revealed a decreasing trend in extracellular DNA release in these samples compared to peroxynitrite-stimulated neutrophils, although this observation was not statistically significant (Supplementary Figure 5a). To analyze, whether our observations are unique to peroxynitrite-induced and NO-induced NETs formation, we also studied the influence of autophagy inhibitors on NETs release upon PMA stimulation. Among late-stage autophagy inhibitors, CQ, but not bafilomycin A1, limited the ability of granulocytes to form web-like structures covering large areas of microscopical slides and slightly diminished DNA release as compared with PMA-stimulated controls (Fig. 2b, c, Supplementary Fig. 5a, b). Furthermore, we observed that 3-MA, but not wortmannin, attenuated PMA-induced NETs formation (Fig. 2b, c, Supplementary Fig. 5a, b).

Both 3-MA and wortmannin have been shown to inhibit class III and class I PI3K, which in turn can regulate the activity of NADPH oxidase. Thus, we have hypothesized that inhibition of RNS-induced NETs release by 3-MA and wortmannin might be caused by the inhibition of ROS production. NBT and DHR 123 oxidation assays have shown a very slight, statistically insignificant increase in ROS production upon RNS stimulation (Fig. 2d, e, Supplementary Fig. 5c, d). Since pre-treatment with PI3K inhibitors significantly decreased ROS production by neutrophils stimulated with SNAP or peroxynitrite, the role of PI3K in the regulation of ROS availability was confirmed (Fig. 2d, e, Supplementary Fig. 5c, d).

Next we investigated the dependence of NO and peroxynitrite-induced NETs release on ROS formation. Prior to the RNS stimulation, we incubated neutrophils with NADPH oxidase inhibitors (apocynin or diphenyleneiodonium—DPI), myeloperoxidase inhibitor (aminobenzoic acid hydrazide—ABAH), or a general ROS scavenger (N-acetylcysteine—NAC, which interferes with the levels of hydrogen peroxide and hydroxyl radical) (Supplementary Fig. 1 presents metabolic targets for ROS/RNS scavengers and inhibitors). All of them inhibited SNAP-induced NETs release, while only ABAH and NAC caused statistically significant inhibition of extracellular DNA release upon peroxynitrite stimulation (Fig. 2f, Supplementary Fig. 5e). Microscopic observations revealed high proportion of decondensed cells and impeded NETs release in samples pretreated with apocynin and stimulated with peroxynitrite, although none of NADPH oxidase inhibitors prevented peroxynitrite-induced NETs release as measured by extracellular DNA content (Fig. 2f, Supplementary Fig. 5e).

Further evidence for the role of NADPH oxidase in NO-induced NETs release came from the studies performed on neutrophils isolated from CGD patients. We observed that the release of NETs by neutrophils isolated from CGD patients was severely abrogated upon 3-h SNAP stimulation. Nevertheless, peroxynitrite-stimulated NETs release was just as efficient as in healthy controls after 3-h stimulation (Fig. 2g, h). Interestingly, in time-course experiments, a delay in peroxynitrite-induced NETs formation was observed in four out of five CGD patients as compared to controls. After 1-h stimulation we observed few or no decondensed cells in samples isolated from CGD patients, when compared with control samples (2 patients, data not shown); after 2-h stimulation in CGD samples, we observed solely the presence of decondensed cells, while in healthy controls, NETs-releasing cells could already be spotted (2 patients, Fig. 2i).

Physiologically, peroxynitrite is formed as a result of simultaneous synthesis of NO and activation of NADPH oxidase yielding superoxide. The differential response of neutrophils from CGD patients to NO and peroxynitrite, as well as a decrease of NETs-inducing potential of NO in the presence of pharmacological NADPH inhibitors, may suggest that peroxynitrite (or its derivatives) mediates NETs release following stimulation with SNAP in healthy controls. Notably, pretreatment with peroxynitrite scavenger, ebselen, decreased NETs formation upon SNAP stimulation, but it failed to completely prevent it (Fig. 2j, Supplementary Fig. 5f). These results suggest that formation of peroxynitrite from NO can only partially explain NETs-inducing properties of SNAP.

### Stimulation of neutrophils with RNS does not induce histone citrullination

Most reports imply that calcium ionophores, but not PMA, cause significant calcium influx and histone citrullination to promote chromatin decondensation [4, 26–29]. In subsequent experiments, we analyzed whether these processes are associated with RNS-induced NETs release. Conversely to CI (calcium ionophore A23187), neither SNAP nor peroxynitrite triggered calcium influx into the cells or histone H3 citrullination (Supplementary Fig. 6), which excluded our initial hypothesis.

### RNS promote translocation of neutrophil elastase into nucleus and cause selective degradation of histones

**Fig. 3:**
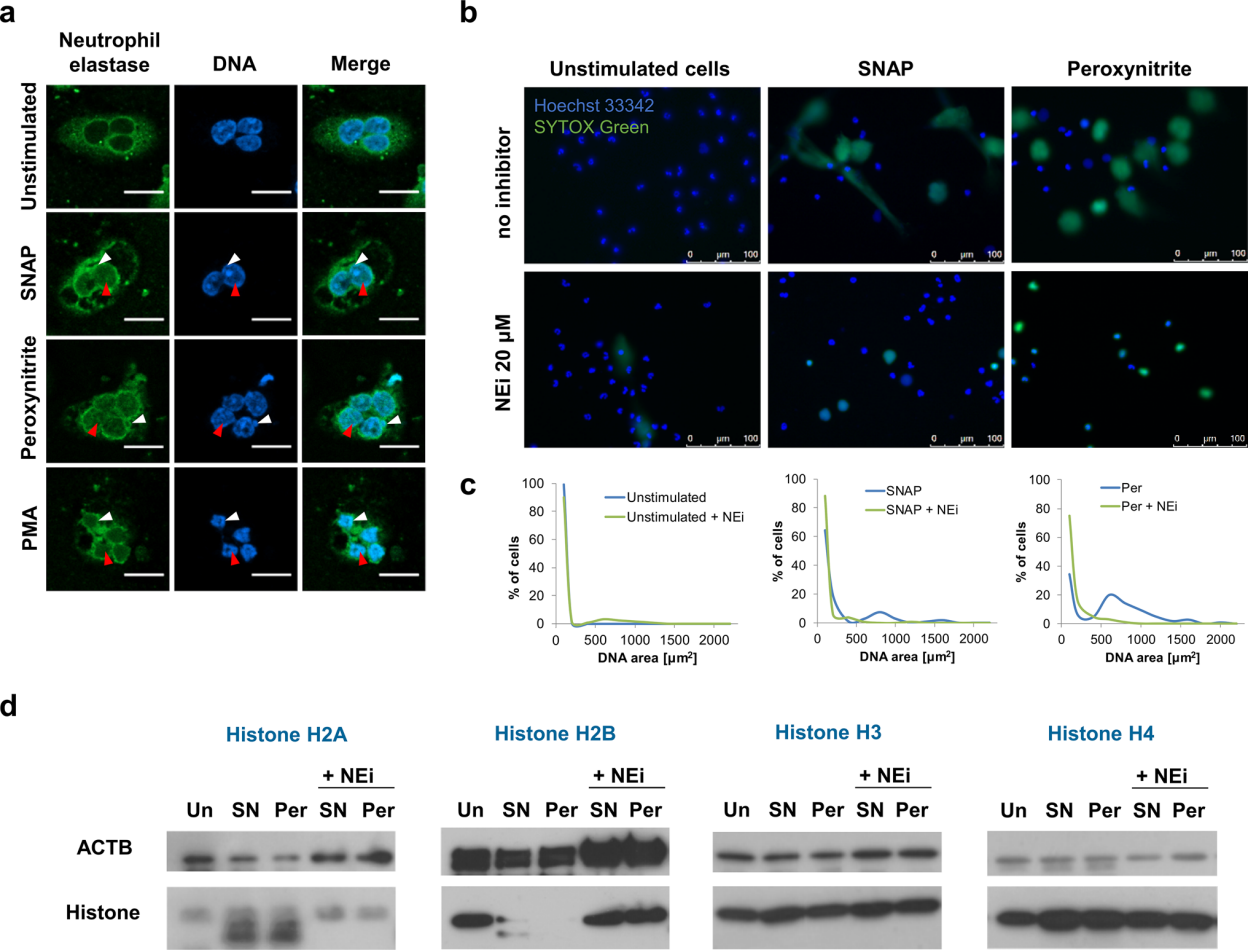
RNS promote translocation of neutrophil elastase into nucleus and cause selective degradation of histones. **a**–**d** Neutrophils were stimulated with 500 µM SNAP (SN), 100 µM peroxynitrite (Per) or 100 nM PMA or left unstimulated (Un) for 60 (**a**) or 180 (**b**–**d**) min. **a** Samples were fixed, stained, and assessed using confocal fluorescent microscopy. Scale bars represent 10 µm. Neutrophil elastase co-localized with DNA at the nuclear membrane (white arrowheads) and within the nucleus (red arrowheads). **b**–**d** Prior to stimulation, neutrophils were preincubated for 30 min with neutrophil elastase inhibitor, GW 311616A (NEi). **b** Unfixed cells were visualized after staining with Hoechst 33342 and SYTOX Green. **c** Areas of SYTOX Green and/or Hoechst 33342-positive objects from **b** were measured using ImageJ software. In each experimental condition, area of at least 100 objects was measured. Results are shown as distribution of percentage of cells over corresponding nuclear area. **b**, **c** Representative results of one out of three experiments using different donors are shown. **d** Degradation of histones was analyzed by western blot. Representative results of one out of three experiments performed using different donors are shown

It was previously shown that at early stages of NETs formation upon PMA stimulation, neutrophil elastase (NE) translocates to the nucleus, where it cleaves histones H2A, H2B, H3, and H4 to enable relaxation of the chromatin structure [10, 26]. We asked whether analogous events can be observed in RNS-stimulated cells. Indeed, using confocal microcopy, we could consecutively observe the colocalization of NE with DNA at the nuclear membrane and within polymorphonuclear nucleus, followed by nuclear decondensation (Fig. 3a, Supplementary Fig. 7).

Further experiments confirmed that NE activity was necessary for RNS-induced nuclear decondensation (Fig. 3b, c, Supplementary Fig. 8). Live imaging allowed us to see inhibition of NETs formation and the preponderance of polymorphonuclear cells in samples pretreated with NE inhibitor (NEi) and stimulated with SNAP or peroxynitrite, contrary to samples not treated with NEi (Fig. 3b, c). Interestingly, in the samples pretreated with NEi and subsequently stimulated with peroxynitrite, most of the cells did not undergo decondensation, but many of them lost the integrity of cell membranes, and were stained both with SYTOX Green and Hoechst 33342 (Fig. 3b). On the contrary, in samples pretreated with NEi and stimulated with SNAP, neutrophils retaining polymorphonuclear shape stained solely with Hoechst 33342 (Fig. 3b). Inhibitory effect of NEi on RNS-induced NETs formation was also confirmed by fluorometric analysis and by immunolabeling of NETs in fixed samples (Supplementary Fig. 8). Further analyses showed that stimulation with RNS resulted in degradation of histones H2A and H2B, but not H3 and H4. Histone degradation upon RNS treatment was prevented by pre-incubation with NEi (Fig. 3d).

### Peroxynitrite, but not SNAP, induces activation of p38 kinase

**Fig. 4:**
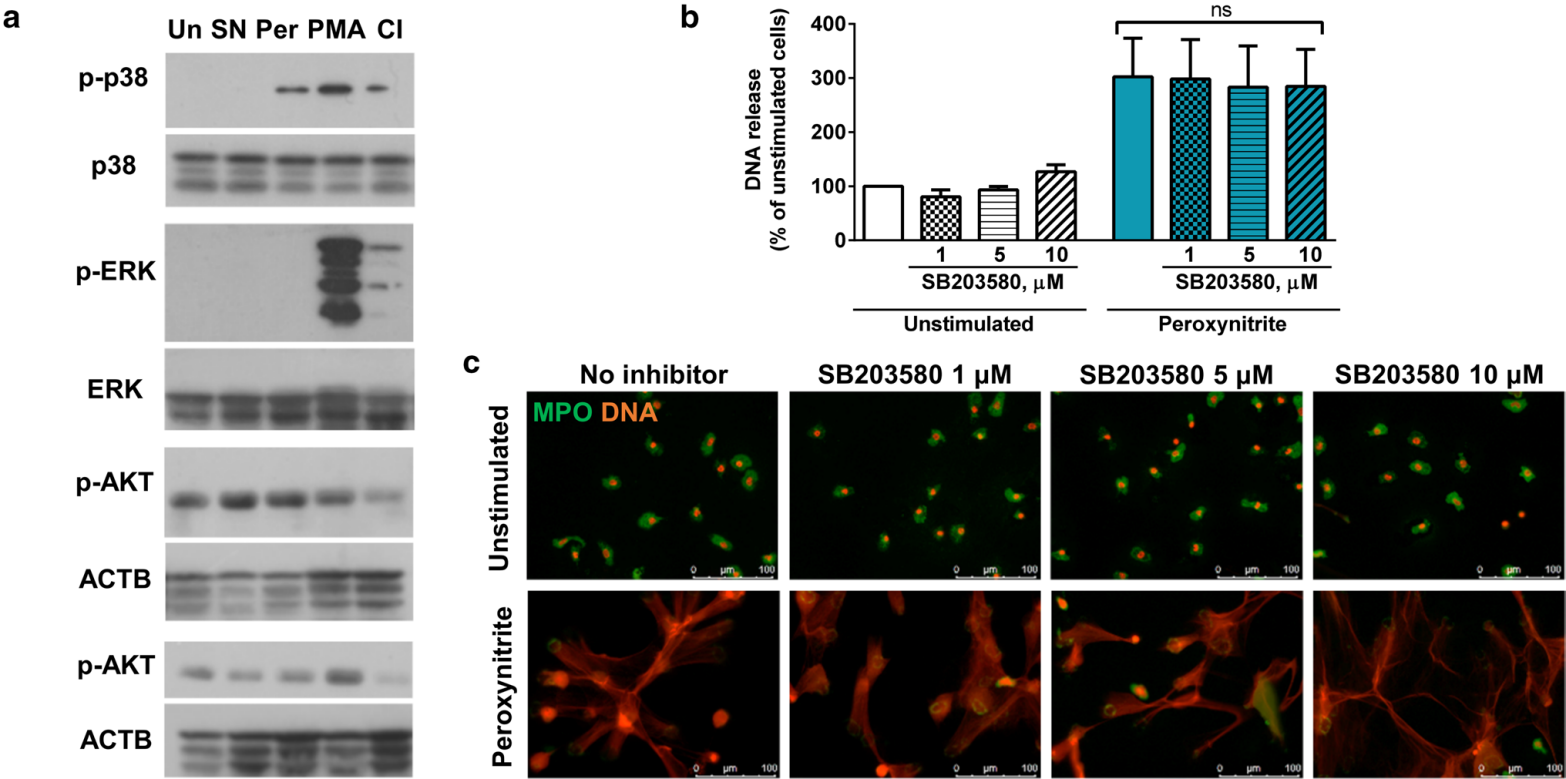
Peroxynitrite, but not SNAP, activates p38 kinase. **a** Cells were stimulated with 500 µM SNAP (SN), 100 µM peroxynitrite (Per), 100 nM PMA, 4 µM calcium ionophore A23187 (CI) or left unstimulated for 30 min and lysed. Activation of signaling kinases was assessed by western blotting. p38, ERK—results representative for at least two experiments using different donors are shown. AKT—4 blood donors were tested, results representative for three blood donors are shown in upper panel, results representative for one blood donor are shown in lower panel. **b**, **c** Neutrophils were pretreated for 30 min with p38 inhibitor, SB203580, and then stimulated with peroxynitrite for 3 h. NETs formation was assessed fluorometrically (**b**) and by fluorescent microscopy (**c**). **b** one-way ANOVA, *n* = 6

Next, we analyzed which signaling cascades are activated in NO and peroxynitrite-stimulated neutrophils by studying phosphorylation of AKT and MAP kinases: p38 and ERK. Peroxynitrite, but not SNAP, activated p38 kinase but no ERK or AKT signaling was observed after stimulation with these compounds (Fig. 4a). We followed up differential kinase-activation patterns induced by various NETs-inducers and we found that stimulation with PMA and CI, strong synthetic NETs inducers, led to phosphorylation of p38 and ERK kinases (Fig. 4a). Only in one out of four blood donors tested, we observed that PMA activated AKT kinase (Fig. 4a, bottom panel). Notably, even though p38 kinase was activated in peroxynitrite-stimulated neutrophils, its activity was not necessary for NETs formation, as shown by the use of p38 inhibitor, SB203580 (Fig. 4b, c).

### RNS potentiate NETs-inducing properties of other stimuli and can be synthesized during formation of NETs

**Fig. 5:**
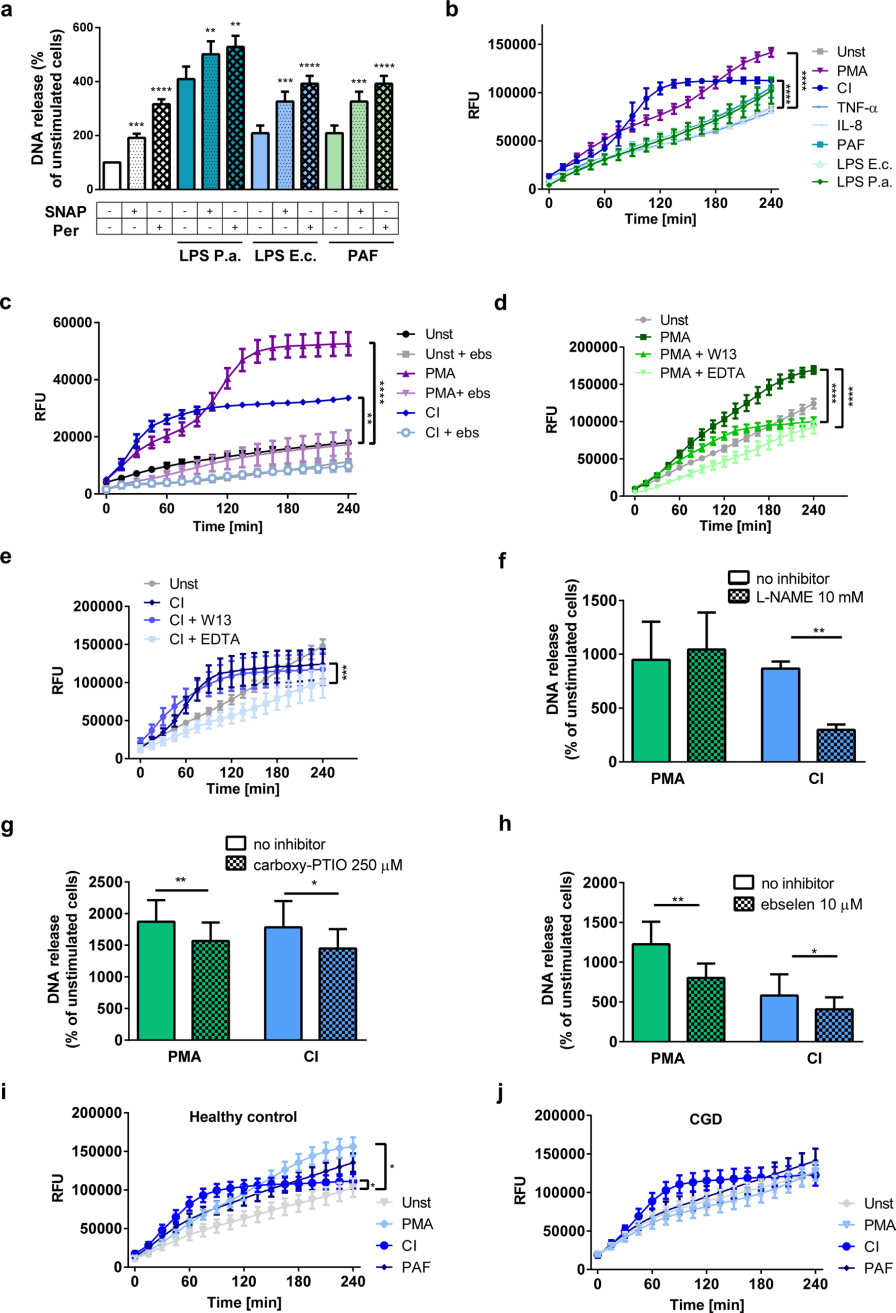
RNS potentiate NETs-inducing properties of other stimuli and can be synthesized during formation of NETs. **a**–**j** Neutrophils were left unstimulated or stimulated to release NETs with 100 nM PMA, 4 µM CI, or with natural inducers of NETs: 2.5 µM platelet activating factor (PAF), 2 µg/ml lipopolysaccharide *E. coli* (LPS E.c.), 10 µg/ml LPS *P.* *aeruginosa* (LPS P.a.), 100 ng/ml tumor necrosis factor α (TNF-α), or 100 ng/ml interleukin 8 (IL-8). In **a**, neutrophils were stimulated with PAF, LPS P.a. or LPS E.c. with or without the addition of RNS: 100 µM SNAP or 100 µM peroxynitrite. **c**–**h** Prior to stimulation, cells were preincubated with 10 µm ebselen (**c**, **h**); 100 µM W13 or 5 mM EDTA (**d**, **e**); 10 mM Nω-nitro-l-arginine methyl ester hydrochloride (L-NAME, **f**) or 250 µM carboxy-PTIO (**g**) for 30 min. **a**, **f**–**h** DNA release was measured fluorometrically after 3-h stimulation. **b**–**e**, **i**, **j** Prior to the stimulation, the neutrophils were loaded with 4-amino-5-methylamino-2′,7′-difluorofluorescein diacetate (**b**, **d**, **e**, **i**, **j**) or DHR 123 (**c**) fluorescent probes; the fluorescence was monitored every 15 min for 4 h post-stimulation. **j** CGD—chronic granulomatous disease patients. **a** Results are shown as means + SEM, *n* = 6, one-way ANOVA with post hoc Dunnett’s test vs. samples not treated with RNS; **b**–**e**, **i**, **j** results are shown as means + SEM from at least three experiments using different donors, two-way ANOVA with post hoc Dunnett’s test; **f**–**h***t* test. (**b**) *n* = 4 for PMA and CI, *n* = 3 for other stimuli, (**c**) *n* = 3, (**d**, **e**, **g**) *n* = 6, (**f**) *n* = 5, (**h**) *n* = 5 for CI and *n* = 6 for PMA (**i**) *n* = 10, (**j**) *n* = 9. *(*p* ≤ 0.05), **(*p* ≤ 0.01), ***(*p* ≤ 0.001), ****(*p* ≤ 0.0001)

We hypothesized that RNS not only stimulate NETs release when used as sole inducers, but also potentiate NETs-inducing properties of physiological stimuli. We incubated neutrophils with PAF or LPS isolated from *E. coli* or *P. aeruginosa*, in the presence or absence of SNAP or peroxynitrite. We found that NETs release in samples co-stimulated with a physiological inducer and SNAP/peroxynitrite was higher than in samples stimulated with any of these stimuli alone (Fig. 5a, Supplementary Fig. 9).

Since exogenously added RNS effectively stimulated neutrophils to release NETs, we next investigated whether endogenous RNS can mediate NETs release induced by other stimuli. Among a variety of NETs-inducers tested (PMA, CI, TNF-α, IL-8, PAF, LPS isolated from *E. coli* or *P. aeruginosa*) (Supplementary Fig. 10), only stimulation with PMA and CI resulted in an increase of NO production by activated neutrophils (Fig. 5b). To detect peroxynitrite generation in these samples, we analyzed the influence of ebselen (peroxynitrite scavenger) on oxidation of DHR 123 probe, which is sensitive to a broad range of oxidants, including peroxynitrite (Fig. 5c). PMA- and CI-induced DHR 123 oxidation was significantly diminished by pre-treatment with ebselen. Calcium depletion with EDTA and/or calmodulin inhibition with W13 prevented NO production after PMA or CI stimulation, suggesting the involvement of neuronal nitric oxide synthase (nNOS, NOS1) and/or endothelial nitric oxide synthase (eNOS, NOS3) in the process (Fig. 5d, e). Since we did not observe any induction of *NOS2* gene in these samples, inducible nitric oxide synthase (NOS2, iNOS) isoform did not seem to be involved (Supplementary Fig. 11).

Next, we analyzed the requirement of RNS for PMA-induced or CI-induced NETs release. Inhibition of NOS with N-nitroarginine methyl ester (L-NAME) and scavenging of NO or peroxynitrite with carboxy-PTIO or ebselen, respectively, decreased NETs release upon PMA and/or CI stimulation, but did not completely prevent it (Fig. 5f–h, Supplementary Fig. 12).

We also investigated, whether impaired production of ROS by neutrophils isolated from CGD patients can be compensated by an increase in RNS release upon stimulation. To this end, we stimulated CGD and control neutrophils (isolated from children and adults) with PMA, CI, or PAF. Consistent with what we observed in experiments with adult blood donors (Fig. 5b), PMA and CI caused significant increase of NO production by neutrophils isolated from healthy controls and PAF showed only a trend toward increased NO production (Fig. 5i). In CGD patients, none of the inducers caused significantly increased NO production; yet we observed a trend toward enhanced NO release upon CI stimulation (Fig. 5j).

## Discussion

Since the discovery of NETs by Zychlinsky’s group in 2004 [1], many researchers were driven by the urge to describe molecular mechanisms underlying NETs release. Although their efforts led to the identification of numerous pathways and molecules implicated in NETs formation, some aspects of this process, such as the role of nitrosative stress, remained unclear. In this study, we thoroughly investigated the process of RNS-induced NETs release and for the first time, we show the NETs-inducing properties of peroxynitrite. We found that the ability of NO and peroxynitrite to induce NETs is NE-dependent and PI3K-dependent and that PI3K inhibition diminished production of ROS by neutrophils. Furthermore, RNS were synthesized during PMA-induced and CI-induced NETs release, as well as scavenging or inhibition of RNS synthesis attenuated NETs induction with these stimuli. Besides being efficient NETs inducers themselves, NO and peroxynitrite potentiated NETs production upon stimulation with physiological inducers.

First evidence on NETs-inducing properties of NO came from the study performed by Patel et al. [16]. They described increased production of free radicals by neutrophils stimulated with NO donors, which resulted in release of NETs [16]. In our studies, we noted only a trend toward increased ROS production by NO-stimulated and peroxynitrite-stimulated granulocytes, although this difference was not statistically significant (Fig. 2d, e, Supplementary Fig. 5c, d). The discrepancies in the observed potential of NO to induce oxidative burst between this and our studies may result from the use of different ROS-sensing probes or concentration of NO donor. Accordingly, the probes differ in their reactivity, specificity, and selectivity, while increasing concentrations of NO exhibit a biphasic influence on ROS production starting from stimulation of oxidative burst up to superoxide scavenging effect [30]. In line with Patel et al. observations, we showed that inhibitors of ROS-producing enzymes, MPO and NADPH oxidase, decreased or completely blocked NETs release upon stimulation with NO (Fig. 2f, Supplementary Fig. 5e) [16]. We further corroborated the importance of ROS for NO-induced NETs release in NADPH oxidase-deficient conditions (CGD patients; Fig. 2g, h). On the other hand, we demonstrated that activity of NADPH oxidase may influence the kinetics, but not the final outcome of peroxynitrite-induced NETs release, as shown after 1-h, 2-h and 3-h stimulation of CGD granulocytes. Still, importance of ROS and oxidative properties of peroxynitrite for induction of NETs have been supported by MPO dependence and inhibition of this process by general anti-oxidant NAC. We observed that ebselen inhibits NETs release upon NO stimulation and that peroxynitrite, but not SNAP, efficiently stimulates NETs release by CGD granulocytes (Fig. 2g, h, j, Supplementary Fig. 5f). Furthermore, NADPH oxidase activity was necessary for NO-induced NETs release, but not for peroxynitrite-induced NETs release, as shown with the use of pharmacological NADPH oxidase inhibitors (DPI and apocynin). Accordingly, it is plausible that not NO itself, but rather its metabolite peroxynitrite and peroxynitrite-derived oxidizing species might be responsible for NETs formation. Indeed, it has been highlighted before that peroxynitrite causes many effects, which have been originally attributed to NO activity [13].

Besides ROS, autophagy also has been implicated in the process of NETs release. Yet, due to a number of conflicting reports, the involvement of autophagy in NETs release is beyond consensus [5, 31, 32]. Most recently it has been suggested that the role of autophagy in NETs release may have been overestimated due to the use of early stage autophagy inhibitors (3-MA and wortmannin) [32, 33]. Results of our study further support this notion. We observed that PI3K inhibitors (3-MA and wortmannin), but not inhibitors of autolysosomal degradation (bafilomycin A1 and CQ), prevented or diminished NO-induced and peroxynitrite-induced NETs formation. This suggests that RNS-induced NETs release depends on PI3K activity, but not on autophagic degradation activity. It needs to be underlined that PI3K inhibitors are not specific to the process of autophagy, but also decrease ROS synthesis. Thus, their inhibitory effect on NETs release might be attributed to their effect on ROS production [32, 33]. Studies by Romao et al. favored the hypothesis that NETs release, at least upon PMA or *C. albicans* stimulation, triggers the activation of PI3K with subsequent ROS production followed by translocation of NE to the nucleus, where it degrades histones [2, 33]. Similar processes might be triggered in RNS-induced neutrophils. Even though stimulation of neutrophils with RNS did not cause a significant increase in ROS production, we have observed that pre-treatment of neutrophils with 3-MA and/or wortmannin resulted in ROS production below basal levels in unstimulated cells (Fig. 2d, e, Supplementary Fig. 5c, d). In accordance with observations by Romao et al., 3-MA had stronger inhibitory activity than wortmannin [33]. Furthermore, we observed that NE translocates to the nucleus in samples stimulated with RNS. Inhibition of NE activity prevented histone degradation and markedly decreased or completely blocked NETs release (Fig. 3).

Surprisingly, we observed that CQ alone induced DNA release by granulocytes. Furthermore, pre-treatment with CQ slightly diminished DNA release and affected the morphology of cells following stimulation with PMA (Fig. 2b, c). Neither of these observations was confirmed with the use of bafilomycin A1, another inhibitor of late-stage autophagy impairing the autophagosome–lysosome fusion. The reason behind these contrasting results remains unclear and requires further investigation. Yet, we speculate that this might be due to off-target effects or diverse mechanisms employed by aforementioned inhibitors (bafilomycin A1 inhibits V-type ATPase, while CQ diffuses into lysosome and increases its pH) [24, 34].

Previous reports suggested that chromatin decondensation is driven by modifications of histones—citrullination and/or degradation of all core histones [2, 10, 26, 35]. In our experimental setting, RNS-induced NETs release occurred without histone citrullination, yet it was associated with degradation of histones H2A and H2B, but not histones H3 and H4 (Fig. 3d, Supplementary Fig. 6). It is interesting to speculate that in RNS-stimulated neutrophils, S-nitrosylation, and protein nitration affect histone structure, rendering H3 and H4 resistant to proteolytic degradation. The role of these posttranslational modifications in NETs release have not been described so far, but it has been proven that RNS induce structural changes, thus affecting the activity of enzymes or receptors expressed by a variety of cells, including neutrophils [13, 30]. Future studies to explore this issue seem warranted.

Since neither NO nor peroxynitrite induced calcium influx or histone citrullination, and their NETs-inducing properties were linked to ROS and histone degradation, we hypothesized that their mode of action was similar to PMA. Yet, these predictions were not fulfilled in experiments revealing differential activation patterns of p38, ERK, and AKT kinases induced by NO, peroxynitrite, and PMA (Fig. 4a). Our observations were concordant with previous reports of Douda et al. [4, 36], who described activation of these kinases in PMA-stimulated neutrophils, but in our experimental conditions, phosphorylation of AKT was donor-specific (Fig. 4a, bottom panel). Conversely, NO failed to activate any of the aforementioned kinases, while peroxynitrite selectively induced p38 kinase activity. Inhibition of p38 kinase did not disrupt NETs formation process upon stimulation with peroxynitrite. Similar findings, i.e. p38 induction which was not required for NETs release, were described for PMA-induced NETs release [4]. It was recently shown that IL-4 abrogates NETs formation via its inhibitory effect on p38 activity [37]. We observed the inability of IL-4 to block peroxynitrite-induced NETs release, which may further underscore the dispensability of p38 activity in this process (unpublished data). Previous reports showed stimulating, inhibitory, or no effects of RNS on activity of AKT and MAP kinases [13, 38, 39]. These contradicting observations can be attributed to differences in experimental setups (e.g. various microenvironments and biphasic, concentration-dependent, influence of RNS on the neutrophil functions) [13, 30, 38, 40]. Thus, specific conditions used in our studies may favor RNS-induced NETs release, independent of MAP and AKT kinases. A differential ability of SNAP and peroxynitrite to induce p38 activation seems surprising, since peroxynitrite is directly derived from NO and it is often believed to mediate most of NO biological effects [13]. This discrepancy could possibly result from lower concentrations of peroxynitrite achieved during spontaneous decomposition of SNAP (yielding NO to react with superoxide), than following a single bolus with a peroxynitrite aqueous solution. Indeed, peroxynitrite effects on p38 activation have been previously identified as concentration dependent [39].

Further evidence that various NETs stimuli may induce distinct activation pathways was provided by experiments performed in NADPH oxidase-deficient setting. CGD granulocytes vigorously responded not only to peroxynitrite, but also to other NETs-inducing molecules: PAF and CI (data not shown). This remains in agreement with the study by Kenny et al. [26] who showed that several NETs-inducing agents, including physiological stimuli of NETs (*C. albicans*, group B *Streptococcus*), are partially or fully independent of NADPH oxidase activity. Instead, NETs release might require mitochondrial or pathogenic microorganisms-derived ROS [4, 26]. Additionally, following induction with PMA, we observed that not only superoxide production but also NO synthesis is defective in CGD patients. (Fig. 5i, j). Similarly, diminished NO production by CGD granulocytes as compared to cells isolated from healthy individuals was previously reported following phagocytic stimulation with *S. aureus* [41].

We have observed that RNS alone efficiently induce NETs, as well as potentiate NETs-inducing properties of physiological NETs stimuli (Figs. 1d, 5a). These results tie well with previous studies by Lim et al. who observed a potentiating effect of NO donor on PMA-induced NETs formation in murine neutrophils [17]. Furthermore, our findings corroborate previous reports on the ability of NO and its derivatives to modulate adhesion, migration, and antimicrobial functions of granulocytes, including chemotaxis and respiratory burst [30, 40].

At the site of infection and inflammation, various RNS are released by multiple types of cells [42]. Among them, neutrophils are major cells infiltrating inflamed tissues and exposed to nitrosative stress. Therefore, it is probable that RNS-induced NETs release we observe ex vivo may also occur in vivo. Notably, the concentrations of peroxynitrite we used throughout our studies reflect concentrations of peroxynitrite achievable in vivo [23, 43]. Furthermore, NETs are involved in the pathogenesis of several disorders and also linked to nitrosative stress, e.g. sepsis, rheumatoid arthritis, and Alzheimer’s disease [2, 13, 14, 27]. Recognition of nitrosative stress as a factor potentiating NETs release is then valuable not only from the scientific point of view, but it may also be clinically relevant implying a potentially druggable target for NETs-related diseases. In the light of successful attempts to target NETs-related pathways in the treatment of systemic lupus erythematosus, cystic fibrosis, or rheumatoid diseases, it might be interesting to study the efficacy of peroxynitrite decomposition catalysts in these conditions [5, 14, 44].

As noted above, granulocytes themselves or surrounding tissues may serve as a source of RNS. The presence of neuronal, endothelial, and inducible NOS isoenzymes has been observed in naïve and/or activated granulocytes [45]. As the activity of the NOS1 and NOS3 is calcium-dependent, limiting influence of EDTA that we observed on PMA- and CI-induced NO release suggests the involvement of constitutive NOS in this process (Fig. 5d, e). Although we failed to observe *NOS2* upregulation in activated granulocytes (Supplementary Fig. 11), still it cannot be excluded that NOS2 is a major isoform implicated in NO production by granulocytes under inflammatory conditions. Current data suggest that changes in *NOS2* expression might be detected after prolonged period of time, e.g. 16-h incubation with cytokines. [46]. Such experimental time frames are usually avoided during in vitro studies as neutrophils can spontaneously undergo apoptosis. However, it is highly probable that the induction of NOS2 is favored in inflammatory settings, when multiple factors elongate the life cycle of granulocytes [31]. Our data also point to the fact that the granulocyte activation pattern might be stimulus-specific and highly diverse. Among a variety of NETs inducers we tested, only PMA and CI increased NO production, but with different kinetics (Fig. 5b). Indeed, there has been some disagreement regarding the ability of granulocytes to produce NO following activation. Some authors failed to observe PMA-induced, CI-induced, or LPS-induced changes in NO release, while others confirmed increased NO production in similar experimental settings [47–49]. There are several plausible explanations for this discrepancy such as different sensitivities of methods applied to detect NO or various bacterial origins of used LPS [50]. Consistent with our observations, the inability of pro-inflammatory cytokines (IL-1 beta or IFN-gamma) to induce NO formation has been reported before [51].

It is worth noting that RNS potentiated NETs release by PAF and LPS, even though these inducers did not increase NO synthesis (Fig. 5a, b). On one hand, it may suggest that co-incubation with RNS activates additional signaling pathways to those initiated by LPS and PAF. This hypothesis is highly likely in the light of previous reports, underscoring the diversity of events triggered by various NETs inducers [4, 5, 26, 52]. Alternatively or complementary, RNS could activate the same molecular events as PAF or LPS. In this scenario, potentiating effect of RNS may simply reflect an increase in the concentration of NETs inducer. It is tempting to speculate that during bacterial infection, surrounding cells produce NO to enhance NETs release by granulocytes stimulated with bacterial components (e.g. LPS).

Finally, our results demonstrated that RNS depletion diminishes PMA-induced and CI-induced NETs formation (Fig. 5f–h). Although NO and peroxynitrite scavengers significantly decreased NETs formation, L-NAME, NOS inhibitor, did not affect PMA-induced release. It can be attributed to the abundance of l-arginine in neutrophils, limiting the activity of competitive NOS inhibitors [46]. Our findings are consistent with data showing that murine neutrophils stimulated with PMA are incapable of releasing NETs after pharmacological NOS inhibition [17]. To our best knowledge, our study is the first to show similar findings in human cells.

Notably, we could not detect increased NO synthesis during NETs release nor NETs-inducing properties of RNS using differentiated HL-60 cell line model. Our observations are in accordance with views expressed by others that HL-60 cell line may only partially resemble functions of peripheral blood neutrophils [18, 53, 54]. The introduction of new, easy-to-transfect, stable cell-line based models would significantly improve the reliability of data regarding neutrophils and NETs biology. Lack of such a model forces researchers to rely on the use of pharmacological inhibitors of different pathways instead, specificity of which has been repeatedly criticized.

Taken together, we provided evidence that involvement of RNS in NETs formation depends on the stimulus and that physiological and synthetic NETs inducers differ in their potential to activate RNS production by neutrophils. Moreover, we showed that NO and its metabolite, peroxynitrite, efficiently triggered NETs release in PI3K-dependent and ROS-dependent manner. Pathways elicited by these two distinct RNS in activated neutrophils were similar, although not identical, and differed from pathways induced by commonly used synthetic stimuli, PMA and CI. These observations correspond well with current consensus; although early studies sought to identify a common mechanism of NETs release, in recent years it has become clear that no universal path can be suggested and multiple diverging mechanisms can lead to a similar outcome [5, 26]. Our observations shed a new light on the possible pathways engaged in NETs formation in various pathological conditions. Consequently, future efforts to explore these issues might open perspectives for novel therapeutic approaches in NETs-related disorders.

### Electronic supplementary material

Below is the link to the electronic supplementary material.

Supplementary material 1 (PDF 7295 kb)

## Abbreviations

3-MA: 3-Methyladenine
ABAH: Aminobenzoic acid hydrazide
AKT: Protein kinase B
CGD: Chronic granulomatous disease
CI: Calcium ionophore A23187
DHR 123: Dihydrorhodamine 123
DPI: Diphenyleneiodonium
ERK: Extracellular signal-regulated kinases
IL: Interleukin
LC3 protein: Light chain 3 protein
L-NAME: N-nitroarginine methyl ester
LPS: Lipopolysaccharide
MAPK: Mitogen-activated protein kinases
MPO: Myeloperoxidase
NAC: N-acetylcysteine
NE: Neutrophil elastase
NEi: Neutrophil elastase inhibitor GW 311616A
NETs: Neutrophil extracellular traps
NBT: Nitroblue tetrazolium
NO: Nitric oxide
NOS: Nitric oxide synthase
PAF: Platelet activating factor
PCR: Polymerase chain reaction
PI3K: Phosphoinositide 3-kinases
PMA: Phorbol 12-myristate 13-acetate
RNS: Reactive nitrogen species
ROS: Reactive oxygen species
SNAP: S-nitroso-N-acetyl-D,L-penicillamine
TNF-α: Tumor necrosis factor α
