# Supplementary material for: Nitric oxide and peroxynitrite trigger and enhance release of neutrophil extracellular traps
Source: Cell Mol Life Sci. 2019 Oct 24;77(15):3059–75. doi: 10.1007/s00018-019-03331-x (PMC7366602; doi:10.1007/s00018-019-03331-x)
Supplement: Supplementary file 1 — Supplementary material 1 (PDF 7295 kb) [file 18_2019_3331_MOESM1_ESM.pdf]

Supplementary information

Nitric oxide and peroxynitrite trigger and enhance release of neutrophil extracellular traps

Cellular and Molecular Life Sciences

Aneta Manda-Handzlik[1,2], Weronika Bystrzycka[1,2], Adrianna Cieloch[1], Eliza Glodkowska-Mrowka[1,3,4,5,6], Ewa Jankowska-Steifer[7], Edyta Heropolitanska-Pliszka[8], Agnieszka Skrobot[1], Angelika Muchowicz[9], Olga Ciepiela[10], Malgorzata Wachowska[1]*, Urszula Demkow[1]*
* These authors contributed equally

1 – Department of Laboratory Medicine and Clinical Immunology of Developmental Age, Medical University of Warsaw, Zwirki i Wigury 63a Street, 02-091 Warsaw, Poland
2 – Postgraduate School of Molecular Medicine, Medical University of Warsaw, Zwirki i Wigury 61 Street, 02-091 Warsaw, Poland
3 – The Finsen Laboratory, Rigshospitalet, Faculty of Health Sciences, University of Copenhagen, Ole Maaloesvej 5, 2200 Copenhagen, Denmark
4 – Biotech Research and Innovation Centre (BRIC), University of Copenhagen, Ole Maaloesvej 5, 2200 Copenhagen, Denmark
5 – Danish Stem Cell Centre (DanStem), Faculty of Health Sciences, University of Copenhagen, Ole Maaloesvej 5, 2200 Copenhagen, Denmark
6 – The Bioinformatics Centre, Department of Biology, University of Copenhagen, Ole Maaloesvej 5, 2200 Copenhagen, Denmark
7 – Department of Histology and Embryology, Medical University of Warsaw, Chalubinskiego 5 Street, 02-004 Warsaw, Poland
8 – Department of Immunology, The Children's Memorial Health Institute, Aleja Dzieci Polskich 20, 04-730 Warsaw, Poland
9 – Department of Immunology, Medical University of Warsaw, Jana Nielubowicza 5 Street, 02-097 Warsaw, Poland
10 – Department of Laboratory Diagnostics, Medical University of Warsaw, Banacha 1a Street, 02-097 Warsaw, Poland

Corresponding author: Malgorzata Wachowska
e-mail: malgorzata.wachowska@wum.edu.pl

Supplementary materials and methods:

Reagents

Roswell Park Memorial Institute (RPMI) 1640 medium, HEPES, micrococcal nuclease (MNase), 4-Amino-5-methylamino-2',7'-difluorofluorescein diacetate (DAF-FM DA), dihydrorhodamine 123 (DHR123), Fluo-3 AM, Fura Red AM, SYTOX Green, SYTOX Orange and Hoechst 33342 were purchased from Thermo Fisher Scientific (Waltham, USA). Wortmannin, bafilomycin A1, platelet activating factor (PAF), carboxy-PTIO, 4-aminobenzoic acid hydrazide (ABAH), W-13, ebselen, phorbol 12-myristate 13-acetate (PMA), S-nitroso-N-acetyl-D,L-penicillamine (SNAP) and sodium peroxynitrite were purchased from Cayman (Ann Arbor, MI, USA). SB203580 was purchased from InvivoGen (San Diego, CA, USA). GW 311616A (neutrophil elastase inhibitor, NEi) was purchased from Axon Medchem (Groningen, The Netherlands) and nitroblue tetrazolium was purchased from Alfa Aesar (Haverhill, MA, USA). Fetal bovine serum (FBS) for HL-60 cell culture was purchased from Biochrom (Berlin, Germany). HL-60 cells (98070106), bovine serum albumin (BSA), Nω-Nitro-L-arginine methyl ester hydrochloride (L-NAME), 3-methyladenine (3-MA), N-acetylcysteine (NAC), calcium ionophore A23187 (CI), SNAP, tumor necrosis factor α (TNF-α), lipopolysaccharide (LPS) isolated from *E. coli* or *P. aeruginosa,* interleukin 8 (IL-8) and all other reagents, unless otherwise stated, were purchased from Sigma Aldrich (St Louis, MO, USA).

Calcium influx assay

To track changes in cytoplasmic calcium concentration, we applied simultaneous double staining with Fura Red AM and Fluo-3 AM dyes, as described in [1] by Demkow et al. Briefly, 2×10^6 neutrophils were loaded with aforementioned calcium indicators (5 µM each, 45 min, 37°C, 5% CO_2, darkness), washed, resuspended in RPMI

medium with HEPES and fluorescence intensity – Fluo-3 vs. time, Fura Red vs. time and Fluo-3/Fura Red ratio vs. time – was monitored with a Cytomics FC 500 Beckman Coulter flow cytometer (Beckmann Coulter, Brea, USA). After 30 seconds of the measurement the acquisition was paused, NETs inducer or RPMI (negative control) was added and the acquisition was continued for ~120 s.

Polymerase chain reaction

Total RNA from 1.4-5.0 × 10^7 neutrophils was isolated using TriPure Isolation Reagent (Roche, Basel, Switzerland). RNA concentration and purity was assessed with NanoDrop One (Thermo Fisher Scientific). Equal amounts of RNA (0.9-1.5 µg, depending on a patient) were reversely transcribed into cDNA with Transcriptor First Strand cDNA Synthesis Kit (Roche) using oligo(dT) and random hexamer primers. Expression of mRNA for target gene: inducible nitric oxide synthase (iNOS, NOS2) was analyzed vs. expression of control genes: actin β (ACTB) and hypoxanthine-phosphoribosyl-transferase (HPRT) by real time polymerase chain reaction (PCR) using pre-designed Taqman probes (Assay IDs: 144412, 143636, 102079; Roche) and LightCycler 480 Probes Master (Roche). PCR reaction was performed in the LightCycler® 480 II (Roche) instrument.

Transmission electron microscopy

The cell pellets were fixed in 2.5% glutaraldehyde in 0.1M phosphate buffer, pH 7.4, for 30 min and postfixed in 1% osmium tetroxide in the same buffer for 1 h. After dehydration in increasing concentrations of ethanol and propylene oxide, the cell pellets were embedded in epoxy resin Poly/Bed 812 (Polysciences Europe GmbH, Eppelheim, Germany), and cut as 70 nm ultrathin sections on a RMC type MTXL ultramicrotome. Sections were contrasted with uranyl acetate and lead citrate and examined using a Jeol 100S electron microscope.

Supplementary figures and figure legends:

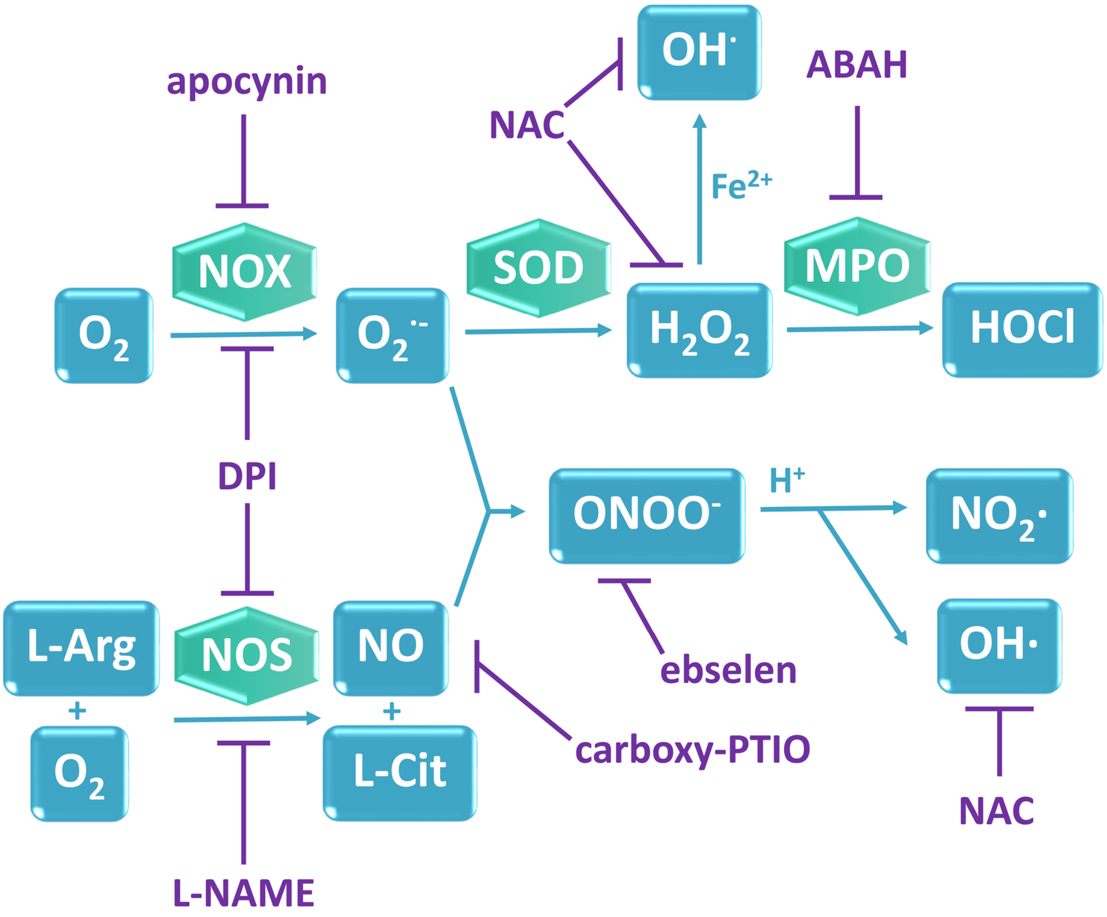

Supplementary Fig. 1 Mechanisms of action of reactive oxygen species/reactive nitrogen species (ROS/RNS) scavengers/inhibitors – schematic representation. NOS – nitric oxide synthase, NOX – NADPH oxidase, SOD – superoxide dismutase, MPO – myeloperoxidase, O_2 – molecular oxygen, L-Arg – L-arginine, NO – nitric oxide, L-Cit – L-citrulline, H_2O_2 – hydrogen peroxide, $O_2^{\cdot -}$ – superoxide, OH· – hydroxyl radical , $ONOO^-$ – peroxynitrite, $NO_2\cdot$ – nitrogen dioxide, HOCl – hypochlorous acid, L-NAME – Nω-Nitro-L-arginine methyl ester hydrochloride, NAC – N-acetylcysteine, DPI – diphenyleneiodonium, ABAH – 4-aminobenzoic acid hydrazide

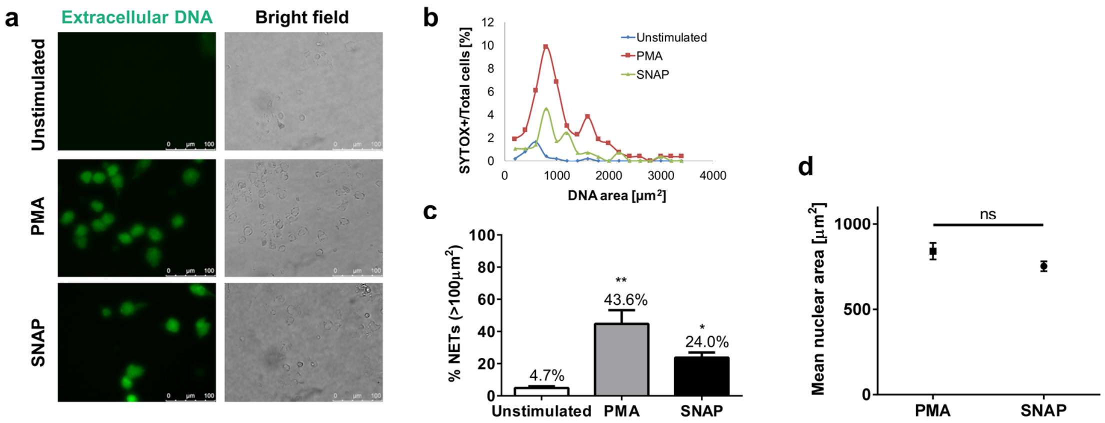

Supplementary Fig. 2 Nitric oxide stimulates release of neutrophil extracellular traps (NETs). **a-d** Neutrophils were stimulated with 500 µM S-nitroso-N-acetyl-D,L-penicillamine (SNAP), 100 nM phorbol 12-myristate 13-acetate (PMA) or left unstimulated for 3 h. After 3 h samples were stained with SYTOX Green, which is impermeant to live cells, and at least 10 images were taken at 40× magnification using fluorescent and transient light. Areas of SYTOX-positive objects were measured using ImageJ software. **a** Representative images of one out of six experiments using different blood donors are shown. **b** Distribution of SYTOX-positive cells percentage over corresponding DNA area is shown, results of one out of six donors tested are shown. **c** Comparison of percentage of NET-releasing cells between samples where NETs were defined as SYTOX+ cells of area larger than 100 μm^2. **d** Comparison of the degree of nuclear decondensation (area of SYTOX+ objects) between SNAP- and PMA-stimulated samples. **c** Results are shown as means + SEM and were analyzed by 1-way ANOVA with post hoc Dunn's test vs. unstimulated cells; $n = 6$. **d** Results are shown as means with SEM out of six experiments using different donors and were analyzed by *t*-test. Statistical significance of the differences is denoted with asterisks: *($p \leq 0.05$), **($p \leq 0.01$)

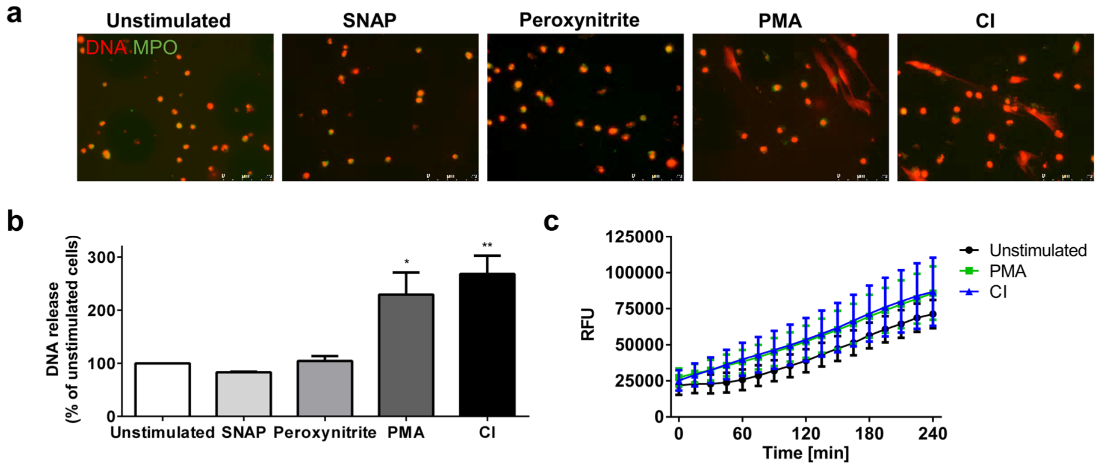

Supplementary Fig. 3 Differentiated HL-60 cells fail to release NETs upon stimulation with reactive nitrogen species (RNS) and do not release NO upon stimulation with PMA or calcium ionophore A23187 (CI). **a-c** HL-60 cells were differentiated with dimethylformamide for 5 days into granulocyte-like cells and then stimulated with 500 µM SNAP, 100 nM PMA, 100 µM peroxynitrite, 4 µM CI or left unstimulated. **a, b** NETs formation was assessed after 3-h stimulation using fluorescent microscopy (**a**) and fluorometrical measurement of DNA release (**b**). **c** The cells were loaded with 4-amino-5-methylamino-2',7'-difluorofluorescein diacetate, stimulated and fluorescence was monitored every 15 min for 4 h post stimulation. **b, c** Means + SEM are shown. **b** 1-way ANOVA with post hoc Dunn's test vs. unstimulated cells. **b** $n = 8$; **c** $n = 4$. *($p \leq 0.05$), **($p \leq 0.01$). *MPO* myeloperoxidase, *RFU* relative fluorescence units

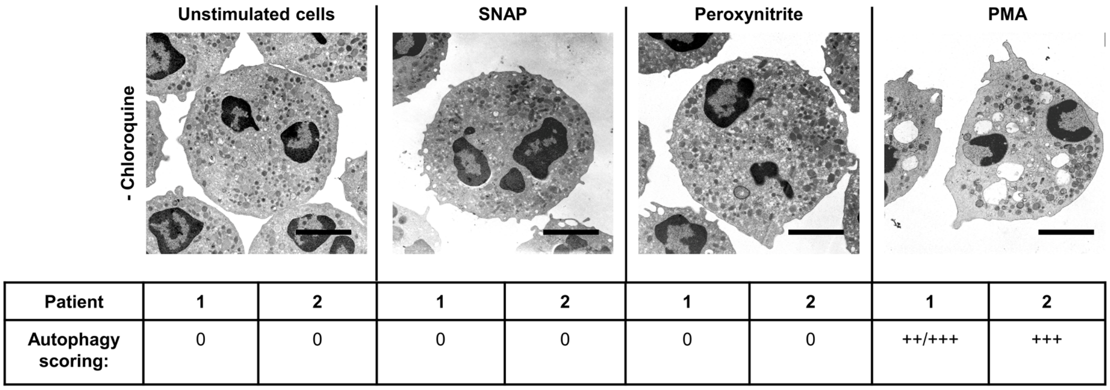

Patient	1	2	1	2	1	2	1	2
Autophagy scoring:	0	0	0	0	0	0	++/+++	+++

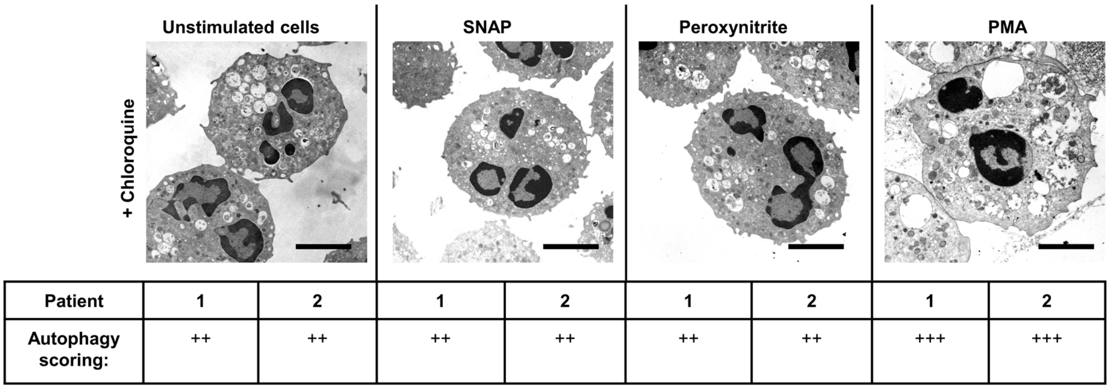

Patient	1	2	1	2	1	2	1	2
Autophagy scoring:	++	++	++	++	++	++	+++	+++

Supplementary Fig. 4 RNS do not induce autophagosome formation. Neutrophils were preincubated with or without 100 µM chloroquine (CQ) for 30 min and then stimulated for 1 h with 500 µM SNAP, 100 µM peroxynitrite or 100 nM PMA. Samples were centrifuged, fixed in 2.5% glutaraldehyde in 0.1M phosphate buffer and further processed for transmission electron microscopy. Images show representative results from one out of two experiments with different donors. Scale bar – 5 µm. Intensity of autophagy process was semi-quantitatively assessed by a trained individual based on a number of autophagosomes per cell, size of the vesicles and the fraction of cells containing the vesicles. For this purpose, 50 cells per condition in each patient was assessed. This resulted in a scoring from 0 to +++ where 0 meant that autophagy was not observed and +++ meant the most intensive process of autophagy. Scorings from two experiments using different donors are written under the corresponding images

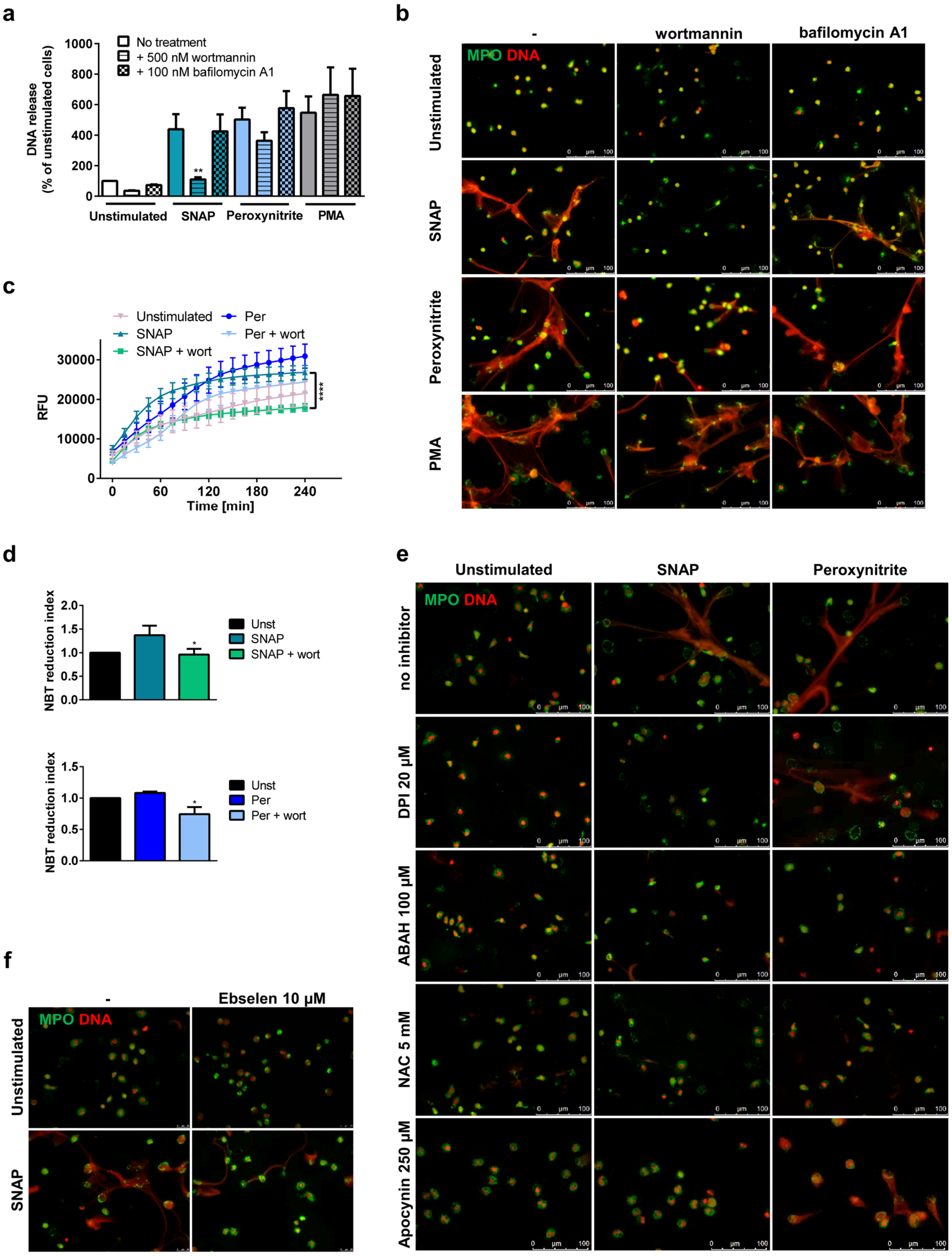

Supplementary Fig. 5 Reactive nitrogen species stimulate NETs in phosphoinositide 3- kinases (PI3K)- and reactive oxygen species (ROS)-dependent manner. **a-f** Neutrophils were preincubated with or without inhibitors: 500 nM wortmannin (wort), 100 nM bafilomycin A1, 100 µM aminobenzoic acid hydrazide (ABAH), 20 µM diphenyleneiodonium (DPI), 5 mM N-acetylcysteine (NAC), 250 µM apocynin, or 10 µM ebselen for 30 min and then stimulated for indicated time with 500 µM SNAP, 100 µM peroxynitrite (Per) or 100 nM PMA. **a, b, e, f** NETs release was analyzed after 3-h stimulation fluorometrically (**a**) and microscopically (**b, e, f**). **c, d** To assess production of ROS, prior to stimulation neutrophils were loaded with dihydrorhodamine 123 and then monitored fluorometrically every 15 min for 4 h post stimulation (**c**) or with nitrotetrazolium blue (NBT) and blue cells containing formazan deposits were counted under the light microscope after 2-h stimulation (**d**). **d** In each

experiment at least 100 cells were analyzed per condition. NBT reduction index was calculated by dividing the percentage of blue cells in each condition by the percentage of blue cells in unstimulated samples. **a, d** Results are shown as means + SEM and were analyzed by one-way ANOVA with post hoc Dunnett's test vs. stimulated cells without inhibitor; $n = 6$. **c** Results are shown as means + SEM out of six experiments with different donors and were analyzed by two-way ANOVA with post hoc Bonferroni's multiple comparisons test. Statistical significance of the differences is denoted with asterisks: *($p \leq 0.05$), **($p \leq 0.01$), ****($p \leq 0.0001$). *Unst* unstimulated cells

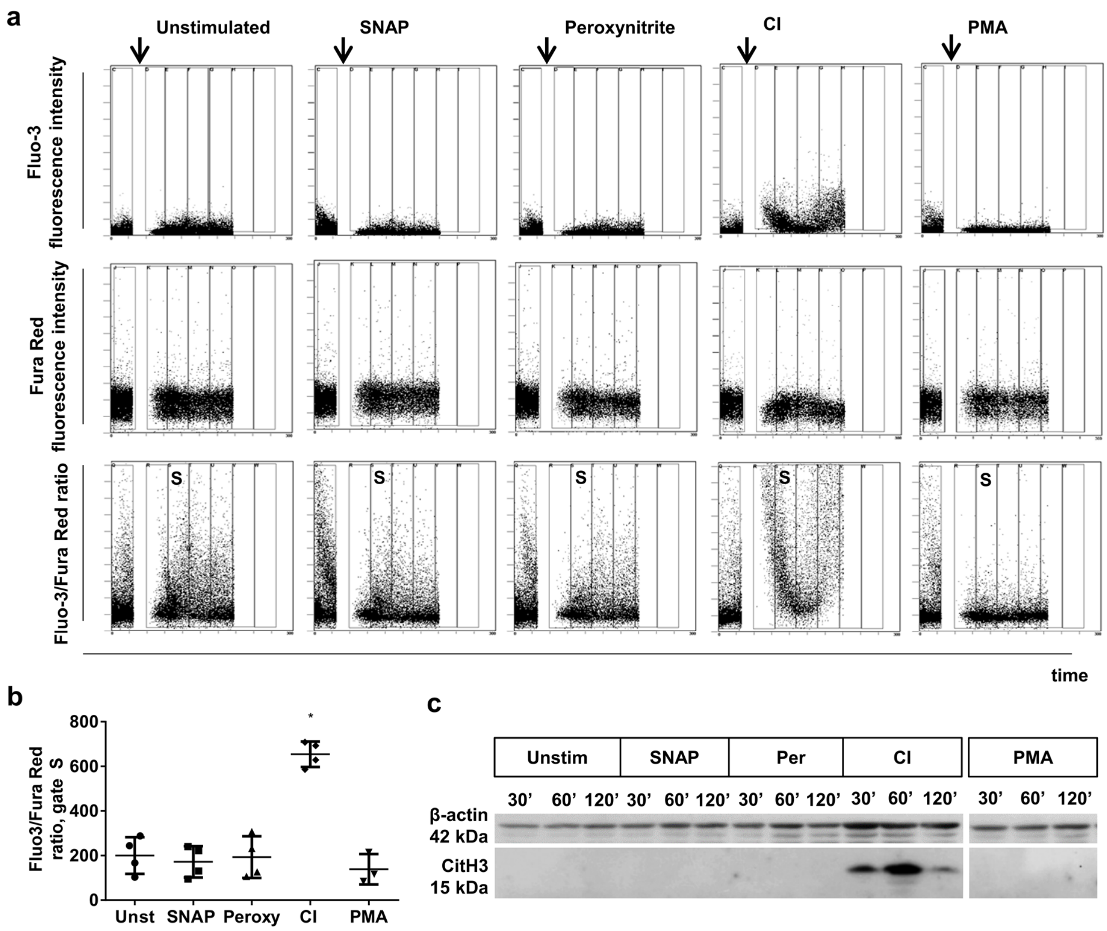

Supplementary Fig. 6 RNS-induced NETs formation is not associated with calcium influx and subsequent histone H3 citrullination. **a, b** Neutrophils were stained with calcium indicators Fluo-3 and Fura Red and fluorescence was measured by flow cytometry. After 30 s the acquisition was paused, cells were stimulated with 500 µM SNAP, 100 µM peroxynitrite, 4 µM CI or 100 nM PMA. RPMI medium was added to control, unstimulated cells. Arrows indicate the moment of stimulation (or RPMI addition) The acquisition was continued for ~120 s. **a** The data show one representative out of four experiments performed with different donors. Each rectangular gate represents 30 s of acquisition. **b** Fluo-3/Fura Red ratio in gate S is presented as the means ± SD with individual data plotted. *($p \leq 0.05$) vs. unstimulated cells, one-way ANOVA with post-hoc Dunn's test, $n = 4$. **c** Cells were stimulated as described above and lysed at indicated timepoints. Histone citrullination was assessed by Western blot. Results of one out of two experiments performed with different donors are shown

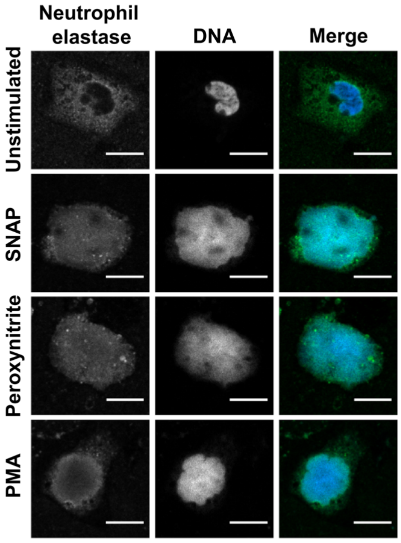

Supplementary Fig. 7 Neutrophil elastase and DNA colocalize in decondensed nuclei of RNS-stimulated neutrophils. Neutrophils were stimulated with 500 µM SNAP, 100 µM peroxynitrite or 100 nM PMA for 120 min. Samples were stained and assessed using confocal fluorescent microscopy. Scale bars represent 10 µm

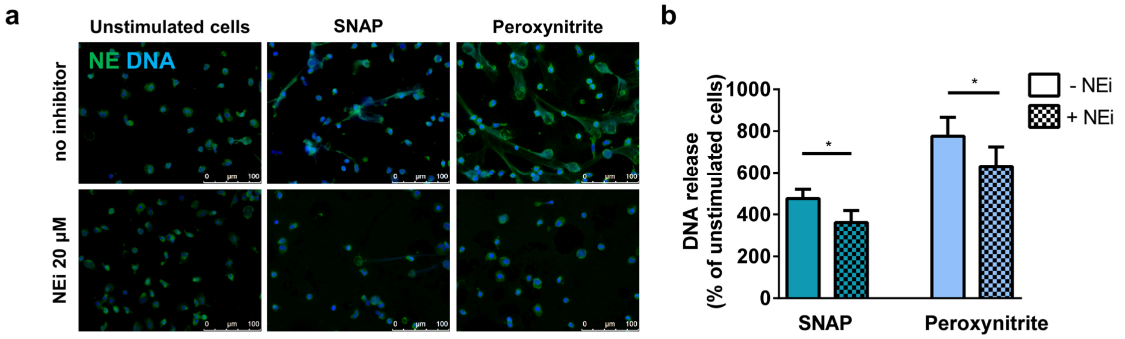

Supplementary Fig. 8 Neutrophil elastase activity is necessary for RNS-induced NETs release. Neutrophils were preincubated for 30 min with or without neutrophil elastase inhibitor (NEi) and then stimulated with 500 µM SNAP, 100 µM peroxynitrite or 100 nM PMA for 3 h. NETs formation was assessed by fluorescent microscopy (**a**) and fluorometry (**b**). **b** Means plus SEM are shown, data were analyzed by *t*-test, $n = 6$, *($p \leq 0.05$)

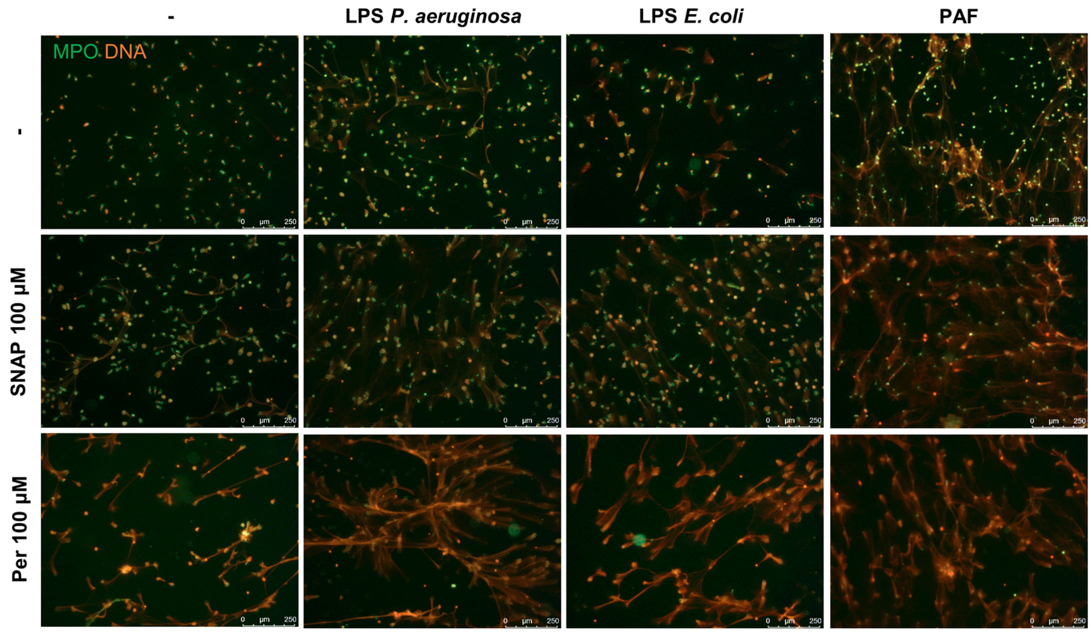

Supplementary Fig. 9 RNS potentiate NETs-inducing properties of other stimuli. Neutrophils were stimulated with natural inducers of NETs: 2.5 µM platelet activating factor (PAF), 2 µg/ml lipopolysaccharide (LPS) isolated from *E.coli* or 10 µg/ml LPS isolated from *P.aeruginosa* with or without addition of RNS: 100 µM SNAP or 100 µM peroxynitrite. NETs release was assessed microscopically after 3-h stimulation. Representative results of one out of three experiments using different donors are shown

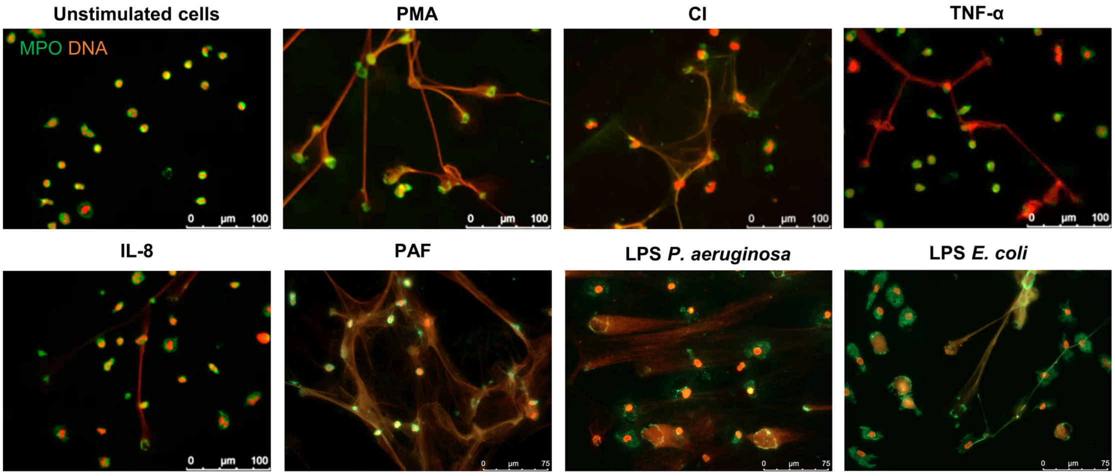

Supplementary Fig. 10 A broad range of inducers stimulate NETs formation. Neutrophils were stimulated with 100 nM PMA, 4 µM CI, 2.5 µM PAF, 2 µg/ml LPS *E.coli*, 10 µg/ml LPS *P.aeruginosa*, 100 ng/ml tumor necrosis factor α (TNF-α), 100 ng/ml interleukin 8 (IL-8) or left unstimulated. NETs release was assessed microscopically after 3-h stimulation. Representative results of one out of three experiments with different donors are shown

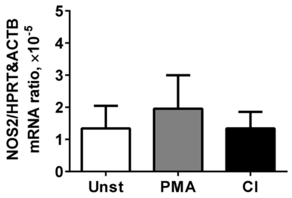

Supplementary Fig. 11 PMA and CI do not induce expression of NOS2 gene. RNA was isolated after 1-h stimulation, reversely transcribed and changes in inducible nitric oxide synthase (NOS2) mRNA expression level vs. expression of control genes (actin β – ACTB, hypoxanthine-phosphoribosyl-transferase – HPRT) were analyzed by real-time PCR

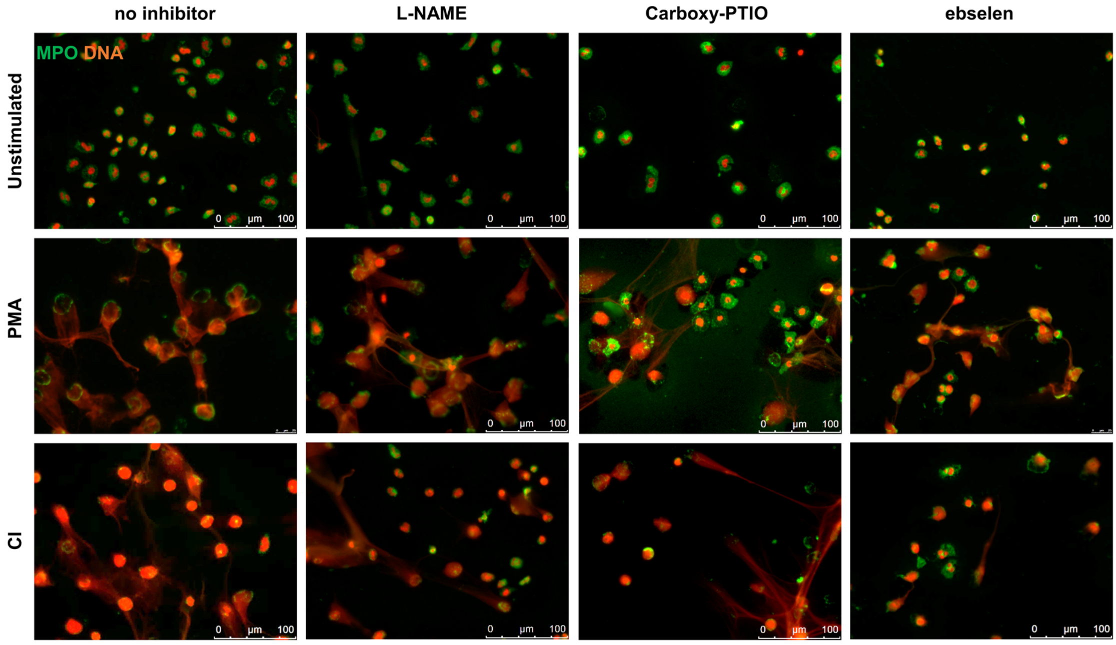

Supplementary Fig. 12 Scavenging of RNS decreases NETs formation upon PMA- and CI-stimulation. Neutrophils were preincubated with or without 10 mM L-NAME, 250 µM carboxy-PTIO or 10 µM ebselen for 30 min and then stimulated with 100 nM PMA, 4 µM CI or left unstimulated. NETs release was assessed microscopically after 3-h stimulation. Representative results of one out of three experiments with different donors are shown

Supplementary tables:
Supplementary Table 1 Characteristics of chronic granulomatous disease (CGD) patients

Patient no.	Age, years	Sex	CGD type	Mutated gene (exact mutation if known); protein encoded by this gene
1	12	Female	Autosomal recessive – AR	NCF1 (c.75_76delGT; p.Tyr26fsX26); p47-phox
2	39	Male	AR	NCF1 (c.75_76delGT; p.Tyr26fsX26); p47-phox
3	27	Female	AR	NCF1 (c.75_76delGT; p.Tyr26fsX26); p47-phox
4	11	Male	X-linked	CYBB; p91-phox
5	15	Female	AR	NCF1 (c.75_76delGT; p.Tyr26fsX26); p47-phox
6	11	Female	AR	NCF1 (c.75_76delGT; p.Tyr26fsX26); p47-phox
7	7	Male	X-linked	CYBB; p91-phox
8	29	Male	not defined - AR or *de novo* CYBB mutation	not defined
9	30	Male	X-linked	CYBB; p91-phox

Supplementary Table 2 Demographic characteristics of healthy controls included in experiments with CGD patients

Healthy control no.	Age, years	Sex
1	9	Male
2	28	Male
3	27	Female
4	6	Male
5	26	Male
6	13	Female
7	16	Female
8	11	Female
9	17	Female

10	26	Male

Supplementary references:

1. Demkow, U., et al., *Kinetics of calcium ion concentration accompanying transduction of signals into neutrophils from diabetic patients and its modification by insulin.* J Physiol Pharmacol, 2009. **60 Suppl 5**: p. 37-40.
